# The Dickkopf1 and FOXM1 positive feedback loop promotes tumor growth in pancreatic and esophageal cancers

**DOI:** 10.1038/s41388-021-01860-z

**Published:** 2021-06-11

**Authors:** Hirokazu Kimura, Ryota Sada, Naoki Takada, Akikazu Harada, Yuichiro Doki, Hidetoshi Eguchi, Hideki Yamamoto, Akira Kikuchi

**Affiliations:** 1 Department of Molecular Biology and Biochemistry, Graduate School of Medicine, Osaka University, Suita, Japan; 2 Department of Gastroenterological Surgery, Graduate School of Medicine, Osaka University, Suita, Japan

**Keywords:** Biochemistry, Pancreatic cancer, Oncogenes, Cell signalling, Prognostic markers

## Abstract

Dickkopf1 (DKK1) is overexpressed in various cancers and promotes cancer cell proliferation by binding to cytoskeleton-associated protein 4 (CKAP4). However, the mechanisms underlying DKK1 expression are poorly understood. RNA sequence analysis revealed that expression of the transcription factor forkhead box M1 (FOXM1) and its target genes concordantly fluctuated with expression of DKK1 in pancreatic ductal adenocarcinoma (PDAC) cells. DKK1 knockdown decreased FOXM1 expression and vice versa in PDAC and esophageal squamous cell carcinoma (ESCC) cells. Inhibition of either the DKK1-CKAP4-AKT pathway or the ERK pathway suppressed FOXM1 expression, and simultaneous inhibition of both pathways showed synergistic effects. A FOXM1 binding site was identified in the 5ʹ-untranslated region of the *DKK1* gene, and its depletion decreased DKK1 expression and cancer cell proliferation. Clinicopathological and database analysis revealed that PDAC and ESCC patients who simultaneously express DKK1 and FOXM1 have a poorer prognosis. Multivariate analysis demonstrated that expression of both DKK1 and FOXM1 is the independent prognostic factor in ESCC patients. Although it has been reported that FOXM1 enhances Wnt signaling, FOXM1 induced DKK1 expression independently of Wnt signaling in PDAC and ESCC cells. These results suggest that DKK1 and FOXM1 create a positive feedback loop to promote cancer cell proliferation.

## Introduction

Dickkopf1 (DKK1) was originally identified as an embryonic head inducer in *Xenopus* embryos and is a secreted protein that antagonizes Wnt signaling [1, 2]. DKK1 is essential for various developmental processes, including anterior-posterior patterning, limb development, somitogenesis, and eye formation [2]. Heterozygous DKK1 deficiency in mice is nonfatal, but these mice have a high bone mass due to increased bone formation [3]. In contrast, transgenic expression of DKK1 causes osteopenia and suppresses cell proliferation in the intestines with architectural degeneration [4, 5]. Thus, DKK1 is involved in many biological phenomena during development and in the adult life of animals.

Of the multiple Wnt signaling pathways, DKK1 inhibits the β-catenin-dependent pathway (β-catenin pathway) [1, 2]. DKK1 induces depalmitoylation and internalization of low-density lipoprotein receptor-related protein 6 (LRP6), a Wnt co-receptor, through a clathrin-mediated route, resulting in removal of LRP6 from the plasma membrane [6–9]. Since expression of DKK1 is directly induced by the activated β-catenin pathway [10], DKK1 creates a negative feedback loop for Wnt signaling.

DKK1 has been considered to act as either tumor suppressor or promoter [2, 11, 12]. As a negative regulator of oncogenic Wnt signaling, DKK1 has been shown to inhibit the growth of various cancer cell lines in vitro and in vivo [2, 11, 13, 14], and DKK1 expression is restrained in some types of cancers due to DNA methylation, polycomb, and micro RNA [15–18]. On the other hand, it has also been shown that DKK1 expression is increased in several cancers [2, 11, 12]. Serum DKK1 levels are also significantly higher in lung, esophageal, and pancreatic cancer patients than in healthy controls [19, 20]. Although the molecular mechanism underlying DKK1-dependent cancer progression was unclear for a long time, it has been recently demonstrated that cytoskeleton-associated protein 4 (CKAP4) is a receptor for DKK1 and that DKK1 activates the phosphoinositide 3-kinase (PI3K)-AKT pathway by binding CKAP4, thereby stimulating cancer cell proliferation [9, 11, 21]. Simultaneous expression of DKK1 and CKAP4 is associated with poor prognosis in pancreatic ductal adenocarcinoma (PDAC), lung adenocarcinoma and squamous cell carcinoma, and esophageal squamous cell carcinoma (ESCC) patients [21–23]. CKAP4 has been recognized as a molecular target for the diagnosis and treatment of pancreatic cancer [24]. The increase in DKK1 expression in cancer may be a result of aberrant activation of Wnt signaling [10]. However, it is unclear why DKK1 expression increases in cancers in which Wnt signaling is not activated aberrantly.

The forkhead box M1 (FOXM1) transcription factor is a member of the forkhead box family of proteins which share a winged-helix DNA-binding domain and are important regulators of animal development and cell differentiation and proliferation [25]. FOXM1 overexpression has been observed in many cancers and actively participates in tumor development by stimulating proliferation [26, 27]. FOXM1 binds to the DNA consensus site C/TAAAC/TA and stimulates the expression of genes involved in cell cycle regulation and cell proliferatio [28, 29].

In this study, we found that DKK1 signaling upregulates FOXM1 expression and that FOXM1 acts as a transcription factor for DKK1 in PDAC and ESCC cells. In addition, we demonstrate that both proteins are frequently and simultaneously expressed in human PDAC and ESCC specimens. These results offer new insight into the mechanism underlying DKK1 overexpression in cancer and suggest that DKK1 and FOXM1 create a positive feedback loop to stimulate cancer cell proliferation.

## Results

### DKK1 and FOXM1 expression correlates in PDAC and ESCC

**Fig. 1:**
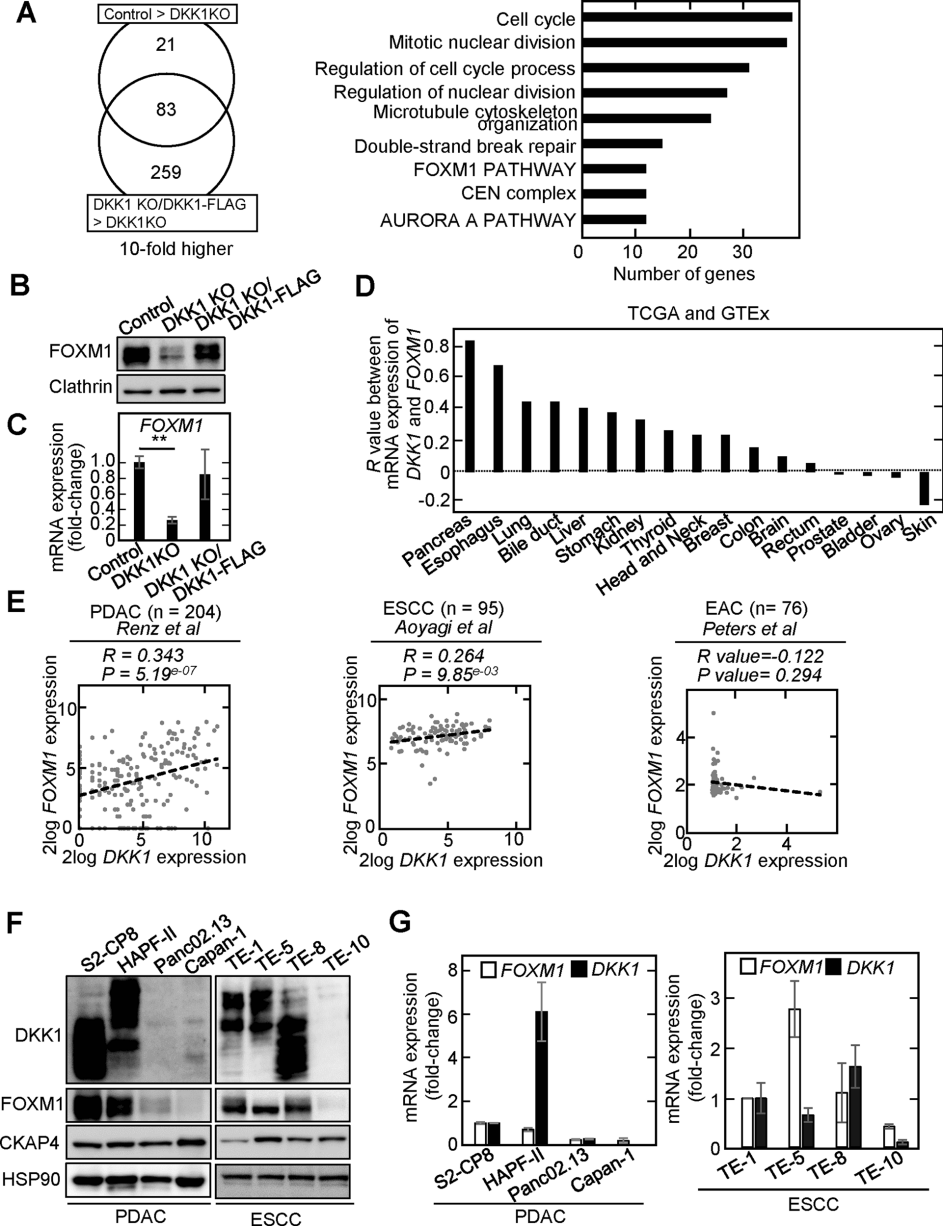
*DKK1* and *FOXM1* expression is correlated in PDAC and ESCC. **A** Workflow of the gene-expression strategy for the identification of DKK1-regulated genes. The Venn diagram summarizes the genes that were more than tenfold overexpressed in control S2-CP8 or S2-CP8/DKK1 KO/DKK1-FLAG cells compared to S2-CP8/DKK1 KO cells (left panel). Enrichment analysis of highly expressed genes in the control S2-CP8 and S2-CP8/DKK1 KO/DKK1-FLAG cells was performed using Metascape (http://metascape.org/gp/index.html#/main/step1) (right panel). **B** and **F** Lysates from S2-CP8 cells used in Fig. 1A (**B**) and various cultured PDAC (**F**, left panel) and ESCC (**F**, right panel) cells were probed with the indicated antibodies. **C** The mRNA level of *FOXM1* in S2-CP8 cells used in Fig. 1A was measured by quantitative RT-PCR and normalized to *GAPDH*. The results are shown as fold-changes compared to control S2-CP8 cells and expressed as means ± SD from three independent experiments. **D** Pearson’s correlation coefficients of the expression of *DKK1* and *FOXM1* mRNAs in normal and cancer tissues from various organs were obtained from TCGA and GTEx datasets. **E** Scatter plot showing the correlation between *DKK1* (X-axis) and *FOXM1* (Y-axis) mRNA expression in PDAC (left panel), ESCC (center panel), and EAC (right panel). The dotted black line indicates linear fit. The data were obtained from R2: Genomics Analysis and Visualization Platform. **G** The mRNA levels of *DKK1* and *FOXM1* in the PDAC (left panel) and ESCC (right panel) cells used in Fig. 1F were measured by quantitative RT-PCR and normalized to *GAPDH*. The results are shown as fold-changes compared to the mRNA level of S2-CP8 or TE-1cells and are expressed as means ± SD from three independent experiments.

To find the uncharacterized signaling pathway regulated downstream of DKK1, two cell lines derived from PDAC S2-CP8 cells were generated: (1) DKK1 knockout cells (DKK1 KO cells) and (2) DKK1 KO cells ectopically expressing DKK1-FLAG (DKK1 rescue cells) (Supplementary Fig. S1A). DKK1 is a glycoprotein and modified with three *N*-linked glycans and two *O*-linked glycans [30]. Therefore, the molecular weights of DKK1 on SDS-PAGE varied and multiple bands of DKK1 were recognized by anti-DKK1 antibody in Western blotting. RNA sequencing analyses were performed using these cells and control S2-CP8 cells. A total of 83 genes were selected based on the criteria that their mRNA levels were decreased more than tenfold in DKK1 KO cells compared to control cells and were increased more than 10-fold in DKK1 rescue cells compared to DKK1 KO cells (Fig. 1A). When enrichment analysis of these genes was performed using Metascape, 9 pathways, including the cell cycle, nuclear division, and cytoskeleton, were shown to be involved (Fig. 1A). These results are consistent with the knowledge that the DKK1-CKAP4 axis promotes cell proliferation through the PI3K-AKT pathway [11, 21]. Among the selected genes, we studied FOXM1 further since it is a transcription factor and master regulator of the cell cycle. In addition, FOXM1 is frequently expressed in various cancers, and its expression is associated with cancer aggressiveness [26, 27]. RNA sequence data revealed that well-known target genes of FOXM1 are reduced by more than 10-fold in the S2-CP8/DKK1 KO cells (Supplementary Fig. S1B).

Consistent with the RNA sequencing data, expression of FOXM1 protein and mRNA was reduced in the S2-CP8/DKK1 KO cells and the downregulation was rescued by DKK1 expression (Fig. 1B, C). mRNAs of *AURKB*, *BIRC5*, and *CENPA*, which are FOXM1 target genes, were also decreased in DKK1 KO cells and their expression was restored by DKK1 expression (Supplementary Fig. S1C). The RNA-sequence dataset obtained from the public domain of The Cancer Genome Atlas (TCGA) and The Genotype-Tissue Expression (GTEx) project revealed that the correlation (*R* value) between the expression of *DKK1* and *FOXM1* mRNAs is higher in pancreatic and esophageal tissues, including tumor and non-tumor regions, compared with other tissues (Fig. 1D). About 90% of pancreatic cancers are PDAC [31], and most of esophageal cancers are either ESCC or esophageal adenocarcinoma (EAC) [32]. The public database revealed a significant correlation between expression of *DKK1* and *FOXM1* mRNAs in the tumor lesions of PDAC [33] and ESCC [34] but not in EAC [35] (Fig. 1E). These prompted us to further examine the relationship of DKK1 and FOXM1 in PDAC and ESCC. By examining different PDAC and ESCC cell lines, we confirmed that DKK1 is highly expressed in cell lines which highly expressed FOXM1 (S2-CP8, HPAF-II, TE-1, TE-5, and TE-8 cells) and that DKK1 expression was low in cell lines with low FOXM1 expression (Panc02.13, Capan-1, and TE-10 cells) at both the protein and mRNA levels (Fig. 1F, G). Thus, expression of DKK1 and FOXM1 is positively correlated in multiple cancer cells.

### The DKK1-CKAP4 pathway is required for FOXM1 expression

**Fig. 2:**
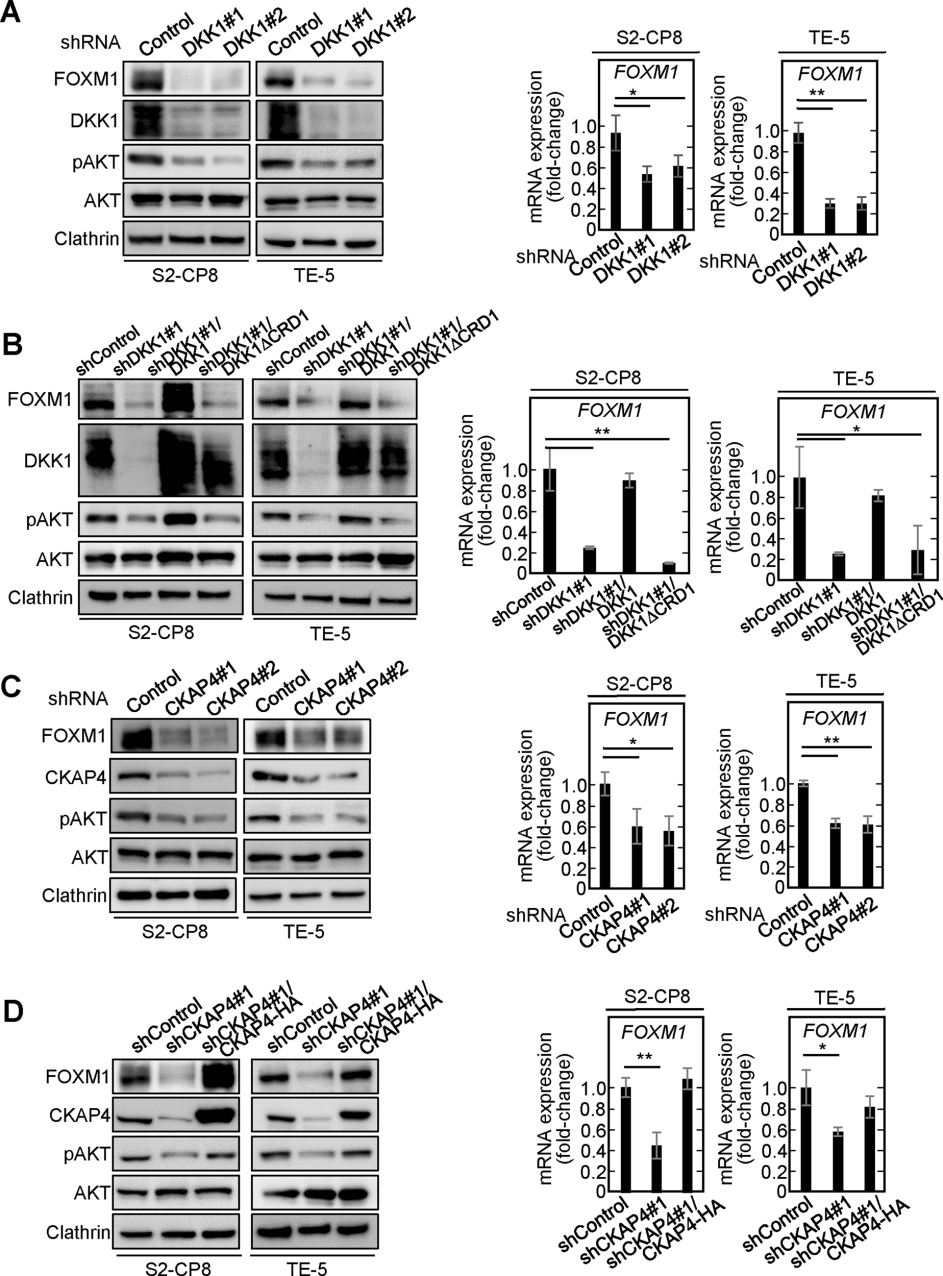
DKK1-CKAP4 signaling is required for FOXM1 expression. Left panels: lysates of S2-CP8 cells and TE-5 cells stably expressing control shRNA or DKK1 shRNAs (**A**), control shRNA, DKK1 shRNA, DKK1 shRNA and DKK1-FLAG, or DKK1 shRNA and DKK1ΔCRD1-FLAG (**B**), control shRNA or CKAP4 shRNAs (**C**), or control shRNA, CKAP4 shRNA, or CKAP4 shRNA and CKAP4-HA (**D**) were probed with the indicated antibodies. Clathrin was used as a loading control. Right panels: the mRNA levels of *FOXM1* in the S2-CP8 cells and TE-5 cells shown in the left panels were measured by quantitative RT-PCR and normalized to *GAPDH*. The results are shown as fold-changes compared to the mRNA level of control shRNA-expressing cells and are expressed as means ± SD from three independent experiments. *P < 0.05; **P < 0.01 (Student’s *t* test).

When DKK1 was knocked down in S2-CP8 and TE-5 cells using two different shRNAs, the levels of FOXM1 protein and mRNA were decreased (Fig. 2A). The decrease in FOXM1 was rescued by expression of wild-type (WT) DKK1 but not by that of DKK1Δcysteine-rich domain (CRD)1, which does not bind to CKAP4 [21] (Fig. 2B). Similarly, CKAP4 knockdown decreased FOXM1 expression in S2-CP8 and TE-5 cells, and the decrease was rescued by restoring CKAP4 expression (Fig. 2C, D). The human *FOXM1* gene has a 10-exon structure and three classes of transcripts—class a, b, and c, are expressed by alternative splicing [28]. FOXM1b and FOXM1c are transcriptional activators that are overexpressed in various types of cancer, whereas FOXM1a is transcriptionally inactive [27]. When the levels of *FOXM1a*, *b*, and *c* mRNA were measured using specific primers, DKK1 knockdown decreased the mRNAs of all three transcripts in S2-CP8 and TE-5 cells (Supplementary Fig. S2A). AKT activation, which was assessed by measuring its phosphorylation, fluctuated in parallel with FOXM1 expression in both DKK1 knockdown (KD) and CKAP4 KD cells (Fig. 2A–D). As shown in Fig. 1F, TE-1 cells expressed DKK1 with low CKAP4 expression, indicating lower activation of the DKK1-CKAP4 axis. Consistently, FOXM1 expression was unchanged by knockdown of DKK1 or CKAP4 in TE-1 cells (Supplementary Fig. S2B and C).

**Fig. 3:**
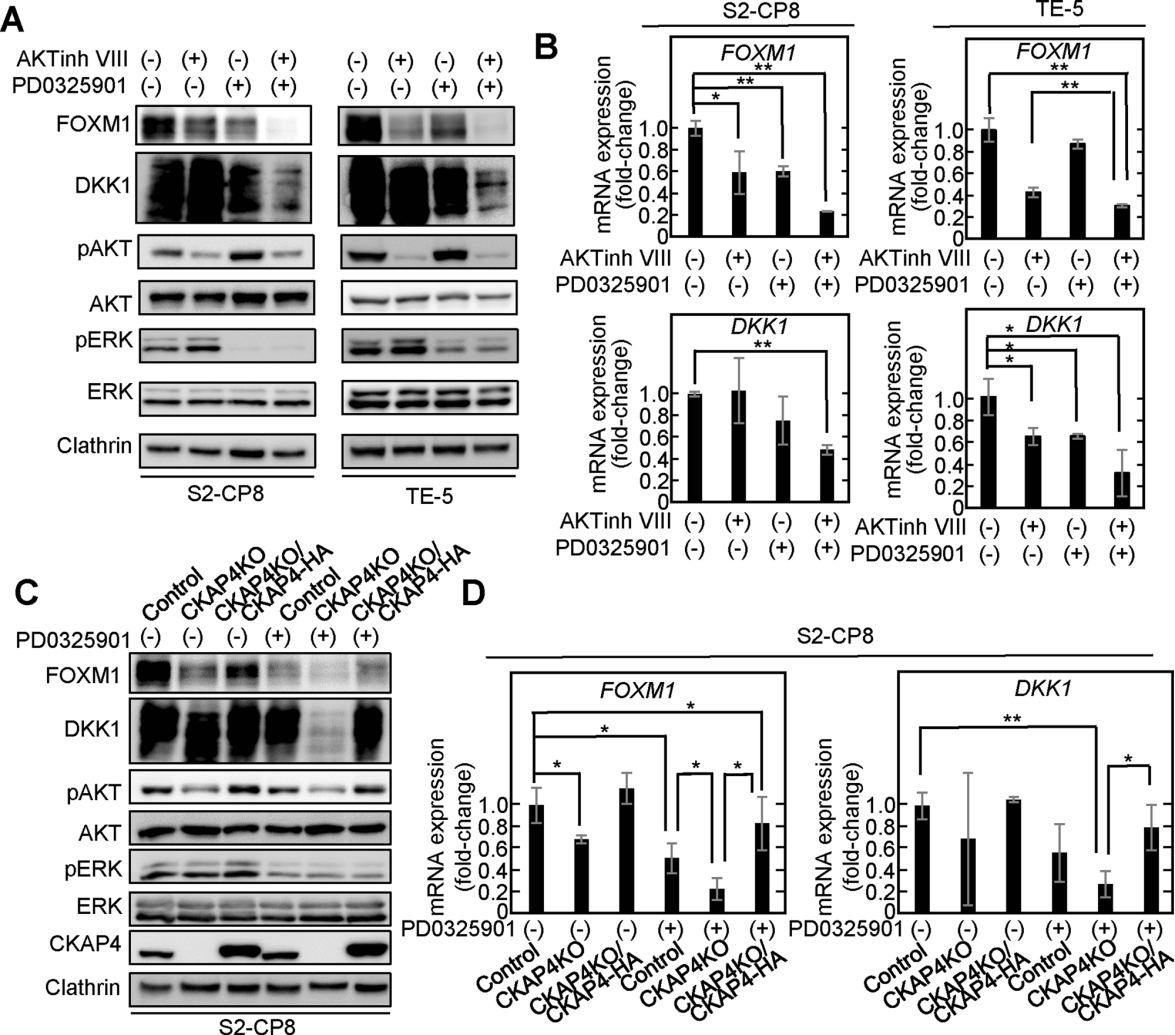
Inhibition of AKT and MEK decreases the expression of FOXM1 and DKK1. **A** S2-CP8 cells were treated with AKT inhibitor VIII (5 μM), PD032590 (5 μM), or both inhibitors for 48 h; and TE-5 cells were treated with AKT inhibitor VIII (50 μM), PD032590 (10 μM), or both inhibitors for 48 h. Lysates were probed with the indicated antibodies. Clathrin was used as a loading control. **B** The mRNA levels of *FOXM1* (top panels) and *DKK1* (bottom panels) in the S2-CP8 cells and TE-5 cells used in Fig. 3A were measured by quantitative RT-PCR and normalized to *UBC*. The results are shown as fold-changes compared to the control cells and are expressed as means ± SD from three independent experiments. **C** Lysates from control S2-CP8 cells, S2-CP8/CKAP4 KO cells, and S2-CP8/CKAP4 KO/CKAP4-HA cells treated with or without PD0325901 (5 μM) for 48 h were probed with the indicated antibodies. Clathrin was used as a loading control. **D** The mRNA levels of *FOXM1* and *DKK1* in the S2-CP8 cells used in Fig. 3C were measured by quantitative RT-PCR and normalized to *GAPDH*. The results are shown as fold-changes compared to the control S2-CP8 cells and are expressed as means ± SD from three independent experiments. *P < 0.05; **P < 0.01 (Student’s *t* test).

ERK directly phosphorylates FOXM1 and stimulates its nuclear translocation and transcriptional activity [36]. AKT increases FOXM1 expression by phosphorylating and inactivating FOXO, which negatively regulates FOXM1 transcription [37]. The AKT inhibitor VIII and the MEK inhibitor PD0325901 suppressed FOXM1 expression in a dose-dependent manner (Supplementary Fig. S3A) and they showed synergistic inhibitory effects when used together in S2-CP8 and TE-5 cells (Fig. 3A, B). Furthermore, it is notable that the simultaneous inhibition of both AKT and MEK decreased DKK1 expression at the protein and mRNA levels (Fig. 3A, B). Similar results were obtained in HPAF-II and TE-8 cells (Supplementary Fig. S3B). PD0325901 suppressed the expression of FOXM1 and DKK1 more strongly in CKAP4 KO S2-CP8 cells than in control cells (Fig. 3C, D). WT CKAP4 expression in CKAP4 KO cells restored FOXM1 and DKK1 expression in the cells treated with PD0325901 (Fig. 3C, D). Taken together, these results suggest that DKK1-CKAP4 signaling is involved in FOXM1 expression via AKT activation and that synergistic signaling with the ERK pathway promotes FOXM1 expression. In addition, these results also suggest that FOXM1 expression is required for DKK1 expression.

### FOXM1 is a transcription factor for DKK1

**Fig. 4:**
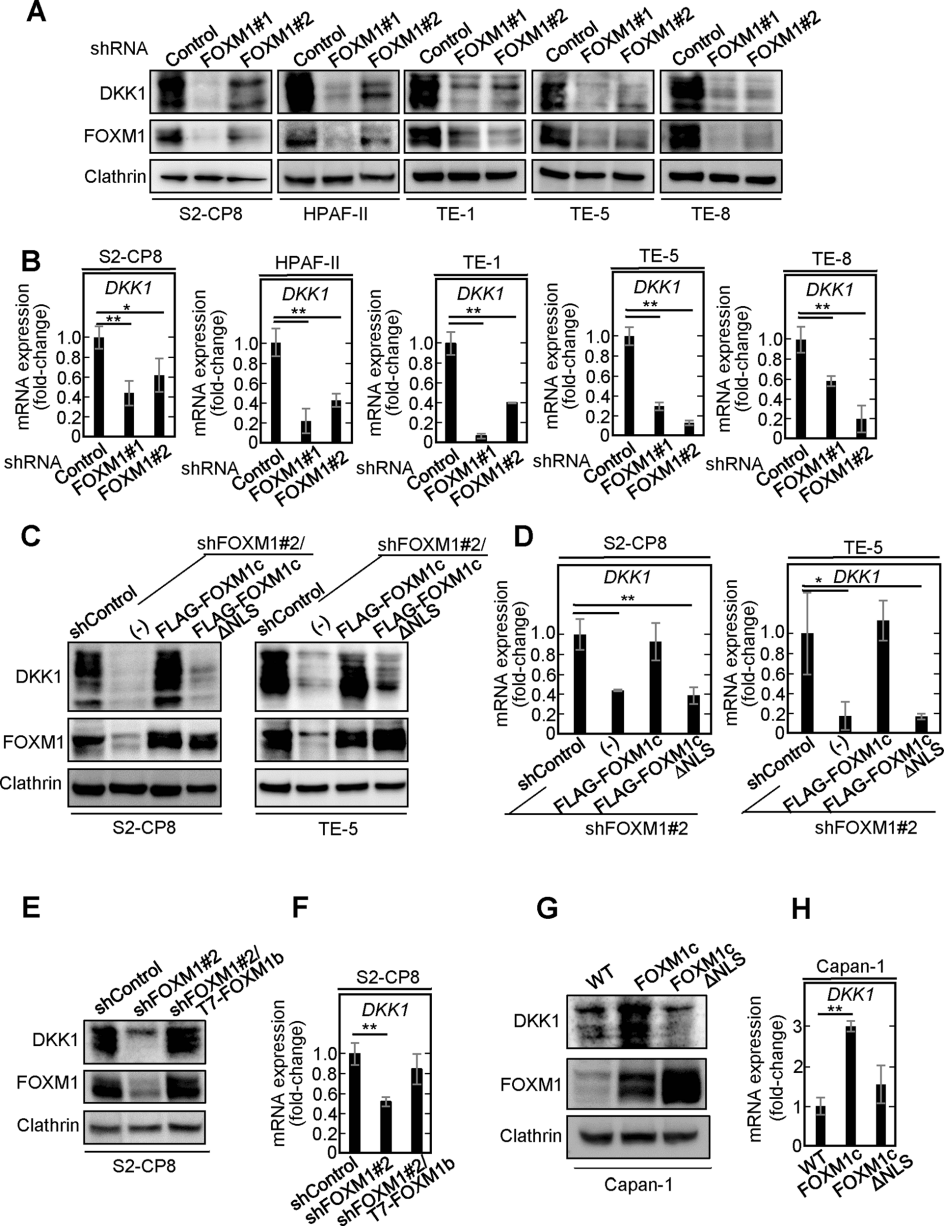
FOXM1 is required for DKK1 expression. **A** Lysates of S2-CP8 cells, HPAF-II cells, TE-1 cells, TE-5 cells, and TE-8 cells stably expressing control shRNA or FOXM1 shRNAs were probed with the indicated antibodies. Clathrin was used as a loading control. **B** The mRNA levels of *DKK1* in various cells used in Fig. 4A were measured by quantitative RT-PCR and normalized to *GAPDH*. The results are shown as fold-changes compared to control shRNA-expressing cells and are expressed as means ± SD from three independent experiments. **C** Lysates of S2-CP8 and TE-5 cells stably expressing control shRNA, or cells stably expressing FOXM1 shRNA with control vector (−), FLAG-FOXM1c, or FLAG-FOXM1cΔNLS were probed with the indicated antibodies. Clathrin was used as a loading control. **D** The mRNA levels of *DKK1* in the S2-CP8 cells and TE-5 cells used in Fig. 4C were measured by quantitative RT-PCR and normalized to *GAPDH*. The results are shown as fold-changes compared to control shRNA expressing cells and are expressed as means ± SD from three independent experiments. **E** and **G** Lysates of S2-CP8 cells stably expressing control shRNA, FOXM1 shRNA, or FOXM1 shRNA and T7-FOXM1b (**E**) and Capan-1 cells stably expressing control vector, FLAG-FOXM1c, or FLAG-FOXM1cΔNLS (**G**) were probed with the indicated antibodies. Clathrin was used as a loading control. **F** and **H** The mRNA levels of *DKK1* in the S2-CP8 cells used in Fig. 4E (**F**) and Capan-1 cells used in Fig. 4G (**H**) were measured by quantitative RT-PCR and normalized to *GAPDH*. The results are shown as fold-changes compared to cells expressing control shRNA or control vector. The results are expressed as means ± SD from three independent experiments. *P < 0.05; **P < 0.01 (Student’s *t* test).

DKK1 expression was reduced at the protein and mRNA levels by two different shRNAs against FOXM1 in S2-CP8, HPAF-II, TE-1, TE-5, and TE-8 cells (Fig. 4A, B). Expression of FOXM1c, but not that of FOXM1cΔNLS, in which the nuclear localization signal (NLS) is deleted, restored the DKK1 expression in S2-CP8/FOXM1 KD and TE-5/FOXM1 KD cells (Fig. 4C, D). FOXM1b expression also rescued the phenotype of the S2-CP8/FOXM1 KD cells (Fig. 4E, F). Furthermore, ectopic expression of FOXM1c, but not that of FOXM1cΔNLS, increased DKK1 expression in Capan-1 cells that otherwise expressed little DKK1 (Fig. 4G, H). Thus, gain- and loss- of-function experiments indicate that FOXM1 positively regulates DKK1 expression.

**Fig. 5:**
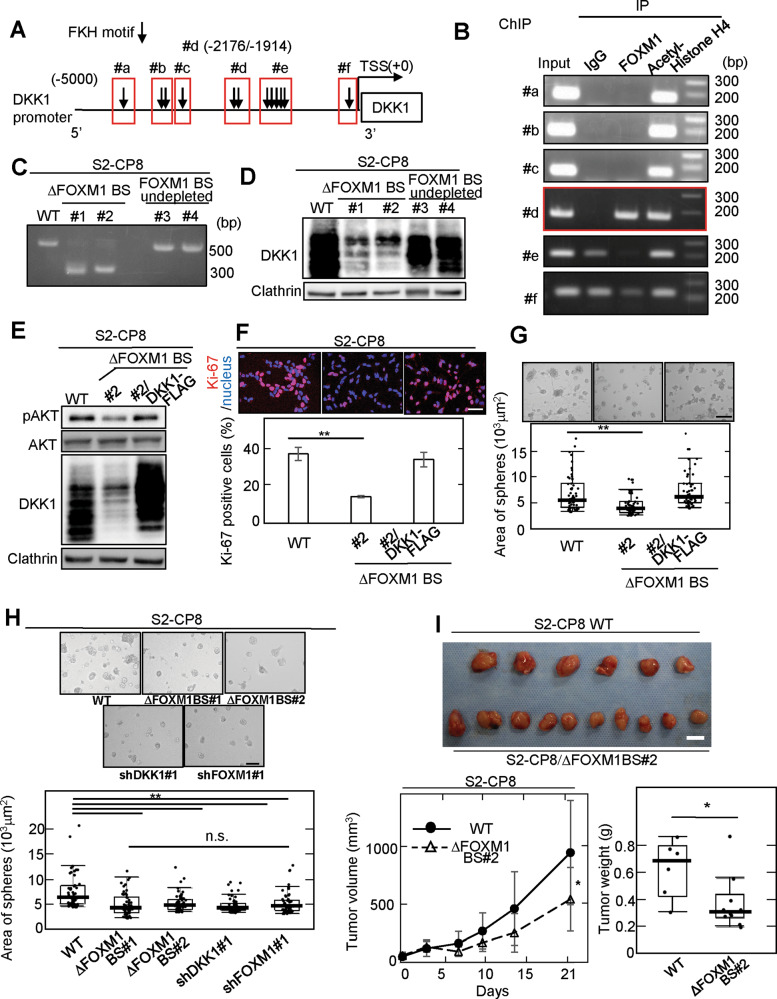
The FOXM1 binding sites of the *DKK1* gene are required for DKK1 expression. **A** Twelve consensus binding motifs of FOXM1 in the *DKK1* genome locus are indicated (black arrows). Putative FOXM1 binding sites were separated into six clusters based on their proximity (surrounded with red frames). **B** ChIP assay was performed using S2-CP8 cells. The chromatin which precipitated with the indicated antibodies was analyzed by PCR with specific primers for each of the putative FOXM1 binding sites. **C** The *DKK1* upstream region was amplified from the genomic DNA of WT (wild type) S2-CP8 cells and S2-CP8/ΔFOXM1 BS cells by PCR. Agarose gel electrophoresis images are shown. WT and FOXM1 binding site deletions are indicated by the presence of a 614 bp and a 359 bp band, respectively. #1 and #2, ΔFOXM1 BS cells; #3 and #4, FOXM1 BS undeleted cells. Lysates of S2-CP8 cells used in Fig. 5C (**D**) and WT S2-CP8 cells, S2-CP8/ΔFOXM1 BS #2 cells, and S2-CP8/ΔFOXM1 BS #2 cells stably expressing DKK1-FLAG (**E**) were probed with the indicated antibodies. **F** Top panels: S2-CP8 cells used in Fig. 5E were stained with anti-Ki-67 antibody (red) and DRAQ5 (blue). Bottom panel: Ki-67-positive cells were calculated and are presented as the percentages of positively stained cells within the total cell population in each field (*n* = 3 fields). The results are presented as means ± SD. Top panels: representative phase contrast images of S2-CP8 cells used in Fig. 5E (**G**) and Figs. 2A, 2C, and 5D (**H**), which were cultured for 5 days in 3D Matrigel, are shown. Bottom panel: the areas of spheres per field (*n* = 3 fields) are plotted as box and whiskers diagrams. Center lines show the medians; box limits indicate the 25th and 75th percentiles as determined by JMP software; whiskers extend 1.5 times the interquartile range from the 25th and 75th percentiles; data points are plotted as dots. *n* = 60 sample points for each cells. **I** WT S2-CP8 cells (*n* = 6) or S2-CP8/ΔFOXM1 BS #2 cells (*n* = 10) were subcutaneously implanted into immunodeficient mice. The volumes of xenograft tumors were measured twice a week for 3 weeks. Top panel: Extirpated xenograft tumors are shown. Bottom panels: Tumor volumes (left panel) and tumor weights (right panel) of WT S2-CP8 cells and S2-CP8/ΔFOXM1 BS #2 cells measured at day 21 are plotted as box and whiskers diagrams. Center lines show the medians; box limits indicate the 25th and 75th percentiles as determined by JMP software; whiskers extend 1.5 times the interquartile range from the 25th and 75th percentiles; data points are plotted as dots. *n* = 6 and 10 sample point. Scale bars, 20 μm (**F**), 100 μm (**G**, **H**), 10 mm (**I**). **P < 0.01 (Student’s *t* test) (**F**), *P < 0.05; **P < 0.01 (Mann–Whitney U test) (**G**–**I**).

To figure out whether FOXM1 directly controls *DKK1* transcription, putative FOXM1 binding sites (BSs) were explored using the UCSC genome browser. Twelve possible FOXM1 binding elements were identified in the area within 5000 bases of the 5ʹ-untranslated region (UTR) of the transcription start site of the human *DKK1* gene, and they were separated into 6 BSs (#a~#f) based on their proximity in the genome (Fig. 5A). Chromatin immunoprecipitation assay coupled with PCR amplification using specific primers revealed that FOXM1 efficiently formed a complex with the site #d (from −2176 to −1914 bases from transcription start site) (Fig. 5B). Next, we knocked out the genomic region including the FOXM1 BS #d in S2-CP8 cells (S2-CP8/FOXM1 BS deletion (ΔFOXM1 BS) cells) using the Crispr-Cas9 system, and two clones of S2-CP8/ΔFOXM1 BS cells (#1 and #2) were established (Fig. 5C and Supplementary Fig. S4A and B). As expected, DKK1 expression was decreased by depleting the FOXM1 BS, whereas FOXM1 BS undepleted control clones (#3 and #4) did not decrease DKK1 expression (Fig. 5D). AKT activation, Ki-67 staining, and the sphere formation were decreased in S2-CP8/ΔFOXM1 BS cells (#2) and DKK1 expression reversed their phenotypes (Fig. 5E–G). The sphere formation of S2-CP8/ΔFOXM1 BS cells (#1 and #2) was reduced to a similar extent as that of S2-CP8 cells in which DKK1 or FOXM1 was knocked down (Fig. 5H). In addition, the volumes and weights of the xenograft tumors derived from S2-CP8/ΔFOXM1 BS cells (#2) were less than those of control tumors (Fig. 5I). These results indicate that FOXM1 directly binds to the site #d of the *DKK1* gene, which stimulates DKK1 expression and cancer cell proliferation.

### DKK1 and FOXM1 are simultaneously expressed in human cancer patients

**Fig. 6:**
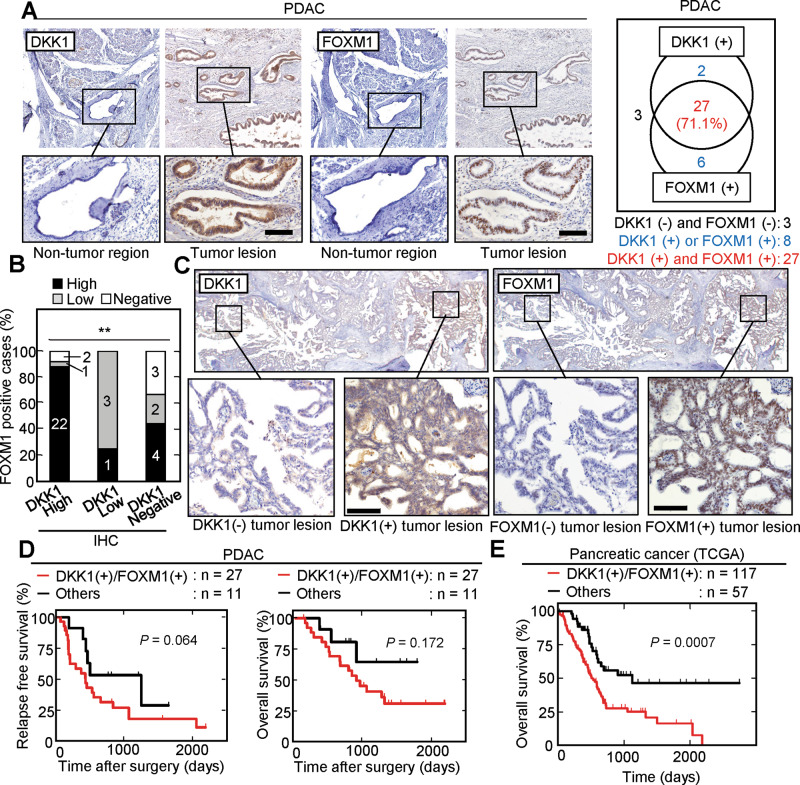
Simultaneous expression of FOXM1 and DKK1 is associated with poor prognosis of PDAC cases. **A** and **C** PDAC tissues (n = 38) were stained with anti-DKK1 or anti-FOXM1 antibody and hematoxylin. Black boxes show enlarged images. Numbers of DKK1- and/or FOXM1-positive (high or low expression) cases are shown in the right panels (**A**). **B** Percentage of FOXM1 high expressing, low expressing, and negative cases in the DKK1 high expressing, low expressing, and negative cases. **D** The relationship between relapse-free survival or overall survival and co-expression of DKK1 and FOXM1 in PDAC patients was analyzed (*n* = 38). Tumors in which positive staining for DKK1 and FOXM1 covered >5% of the total area were classified as DKK1 (+) / FOXM1 (+) (*n* = 27; 71.1%). **E** TCGA mRNA expression levels and clinical outcome data for pancreatic cancer patients were retrieved from OncoLnc (http://www.oncolnc.org). Positive expression of *FOXM1* and *DKK1* (DKK1(+)/FOXM1(+)) group were classified based on the top 80% of mRNA values of *FOMX1* or *DKK1*. **P < 0.01 (Pearson’s chi-square test); Scale bars, 100 µm (**A** and **C**). The data were analyzed using Kaplan–Meier survival curves, and the Generalized Wilcoxon test was used for statistical analysis (**D** and **E**).

Immunohistochemistry (IHC) staining for DKK1 and FOXM1 in PDAC tissues (38 cases) was performed. The cases were classified into three groups based on the degree of tumor areas positive for IHC staining, <5% (negative), 5-20% (low expression), and >20% (high expression). Those with ≧5% staining were considered positive. Positive DKK1 and FOXM1 staining were seen in 29/38 (76.3%) and 33/38 (86.8%) cases, respectively, while both proteins were minimally detected in the non-tumor regions of pancreatic ducts under our staining conditions (Fig. 6A). As a validation of anti-DKK1 and anti-FOXM1 antibodies used in this study, the IHC assay using isotype control IgG was performed, and the staining was rarely observed (Supplementary Fig. S5A). Positivity of DKK1 and both DKK1 and FOXM1 staining was significantly associated with perineural invasion (*P* = 0.018 and 0.045, respectively) (Supplemental Table 1), and 27/38 (71.1%) cases were positive for both DKK1 and FOXM1 in serial sections (Fig. 6A). The correlation between the ratios of tumor lesions with positive DKK1 and FOXM1 staining was confirmed (Fig. 6B), namely tumor lesions which highly expressed DKK1 were also positive for FOXM1, and tumor lesions not expressing DKK1 were negative for FOXM1 (Fig. 6C).

The relapse-free survival rate tended to be lower in PDAC cases with the expression of both DKK1 and FOXM1 compared to other cases (cases positive for either DKK1 or FOXM1 alone or negative for both) (median relapse-free survival 419 days for both DKK1 and FOXM1 cases; 1247 days for other cases; *P* = 0.064). The analyses concerning of overall survival rate also showed a similar tendency (Fig. 6D). A small number of cases might have impaired our statistic power; nevertheless, the TCGA dataset indicated a significant correlation between the expression of *FOXM1* and *DKK1* mRNAs in PDAC cases (Supplementary Fig. S5B). When cases were separated into *DKK1* and *FOXM1* positive expression (DKK1(+)/FOXM1(+)) group and others based on the top 80% of mRNA values of *FOMX1* or *DKK1*, 117 of 174 pancreatic cancer cases (67.2%) were classified as DKK1(+)/FOXM1(+) group, of which positive ratio was similar to that of IHC study for DKK1 and FOXM1 (Fig. 6A), and 57 cases (32.8%) were classified as others (Fig. 6E). Overall survival was significantly reduced in the DKK1(+)/FOXM1(+) group compared to others (*P* = 0.0007) (Fig. 6E).

**Fig. 7:**
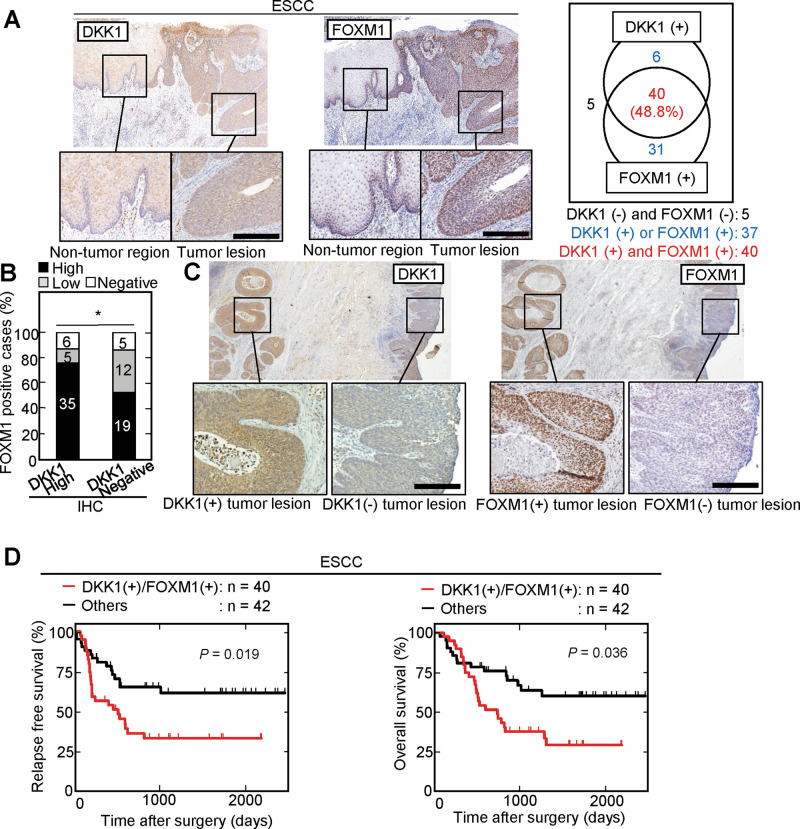
Simultaneous expression of FOXM1 and DKK1 is associated with poor prognosis of ESCC cases. **A** and **C** ESCC tissues (*n* = 82) were stained with anti-DKK1 or anti-FOXM1 antibody and hematoxylin. Black boxes show enlarged images. Numbers of DKK1- and/or FOXM1-positive (high or low expression) cases are shown in the right panels (**A**). **B** Percentage of FOXM1 high expressing, low expressing, and negative cases in the DKK1 high expressing and negative cases. **D** The relationship between relapse-free survival or overall survival and co-expression of DKK1 and FOXM1 in ESCC patients was analyzed (*n* = 82). Tumors in which positive staining for DKK1 and FOXM1 covered >5% of the total area were classified as DKK1 (+) / FOXM1(+) (*n* = 40; 48.8%). *P < 0.05 (Pearson’s chi-square test); Scale bars, 100 µm (**A** and **C**). The data were analyzed using Kaplan–Meier survival curves, and the Generalized Wilcoxon test was used for statistical analysis (**D**).

We also used IHC to measure DKK1 and FOXM1 expression in ESCC (82 cases). DKK1- or FOXM1-positive cells were only minimally detected in the non-tumor epithelium, whereas tumor lesions showed clear staining for DKK1 and FOXM1 (Fig. 7A). In total, 46/82 (56.1%) and 71/82 (86.6%) ESCC cases were positive for DKK1 and FOXM1 expression, respectively. Both DKK1 and FOXM1 were present in serial sections from 40/82 (48.8%) cases. The correlation between the ratios of tumor lesions stained with DKK1 and FOXM1 was confirmed (Fig. 7B), namely an ESCC tumor lesion which highly expressed DKK1 was also positive for FOXM1, and a tumor lesion which did not express DKK1 was negative for FOXM1 (Fig. 7C).

Positive DKK1 staining was significantly associated with lymph node metastasis (*P* = 0.04) (Supplementary Table 2). Both the relapse-free survival rate and overall survival rate were poor in DKK1 and FOXM1 double positive cases (*P* = 0.019 and *P* = 0.036, respectively) (Fig. 7D). Univariate analysis of ESCC cases demonstrates that pN1-3, DKK1 positivity, and DKK1 and FOXM1 double positivity are associated with shorter relapse-free survival (Supplementary Table 3). Multivariate analysis identified that being both DKK1 and FOXM1 positive was an independent prognostic factor (*P* = 0.036) (Supplementary Table 4). Taken together, these results indicate that the simultaneous expression of DKK1 and FOXM1 is associated with poor prognosis in PDAC and ESCC.

### DKK1 expression is induced by FOXM1 independently of Wnt signaling

**Fig. 8:**
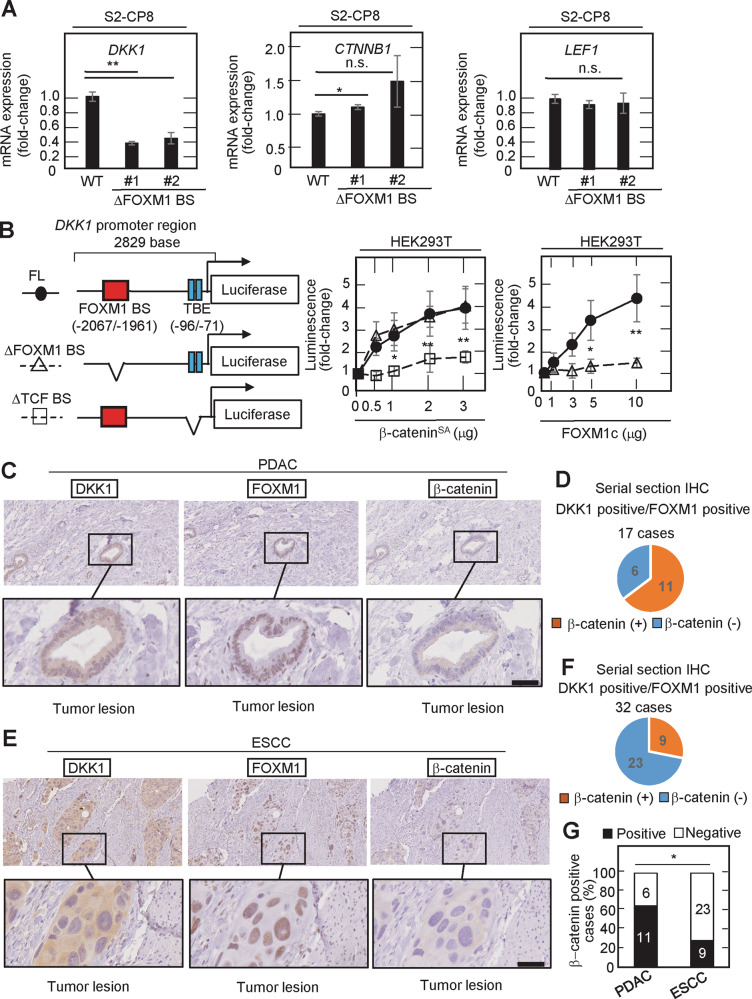
FOXM1 induces DKK1 expression independently of Wnt signaling. **A** The mRNA levels of *DKK1*, *CTNNB1*, and *LEF1* in WT S2-CP8 or S2-CP8/ΔFOXM1 BS #1 and #2 cells were measured by quantitative RT-PCR and normalized to *GAPDH*. The results are shown as fold-changes compared to WT S2-CP8 cells and are expressed as means ± SD from three independent experiments. **B** HEK293T cells were transfected with the indicated amounts of HA-β-catenin^SA^ expression vector (middle panel) or FLAG-FOXM1c expression vector (right panel) and the indicated reporter constructs shown in the left panels, and luciferase activities were measured. The results are shown as fold-changes compared to cells without β-catenin^SA^ or FOXM1c expression. Luciferase activities of cells transfected with the reporter construct containing the full-length *DKK1* upstream sequence (FL) are indicated by the solid line and closed circles, those with the mutant *DKK1* upstream sequence in which the FOXM1-binding site was deleted (ΔFOXM1 BS) are indicated by the dotted line and open triangle, and those with the mutant *DKK1* upstream sequence in which the TCF-binding site was deleted (ΔTCF BS) are indicated by the dotted line and open square. The results are expressed as means ± SD from three independent experiments. **C** and **E** Serial sections of DKK1 and FOXM1 double positive PDAC tissues (*n* = 17) (**C**) and ESCC tissues (*n* = 32) (**E**) were stained with anti-DKK1, anti-FOXM1, or anti-β-catenin antibody and hematoxylin. Black boxes show enlarged images of tumor lesions that are positive for DKK1 and FOXM1 and negative for β-catenin. Numbers of β-catenin positive or negative cases are shown in a pie chart (**D** and **F**). β-catenin positivity in DKK1 and FOXM1 double positive PDAC and ESCC tissues is shown in the bar graph (**G**) and was statistically analyzed using the chi-square test. *P < 0.05; **P < 0.01 (Student’s *t* test); scale bars, 50 µm (**C** and **E**).

It has been reported that DKK1 is a direct target of Wnt signaling in various cells [10] and that FOXM1 binds directly to β-catenin and promotes its nuclear localization and β-catenin-dependent transcriptional activity in glioma cells [38]. Therefore, we investigated whether FOXM1 induces DKK1 expression independently of Wnt signaling in PDAC and ESCC cells. β-Catenin was primarily observed in the cytoplasm and nucleus of S2-CP8 and HPAF-II cells, which is the hallmark of Wnt signaling activation, whereas it was present in the plasma membrane of TE-5 and TE-8 cells (Supplementary Fig. S6A). Knockdown of *CTNNB1*, the β-catenin gene, decreased the mRNA level of *AXIN2*, a well-known target of Wnt signaling, in S2-CP8 and HPAF-II cells but not in TE-5 cells (Supplementary Fig. S6B). This suggests that Wnt signaling is activated in S2-CP8 and HPAF-II cells, but not in TE-5 and TE-8 cells. In addition, *CTNNB1* knockdown inhibited DKK1 expression in S2-CP8 cells and FOXM1 formed a complex with β-catenin in S2-CP8 cells, but these phenotypes were not observed in HPAF-II, TE-5, or TE-8 cells (Supplementary Fig. S6B and C). Therefore, it is likely that DKK1 is expressed independently of Wnt signaling in HPAF-II, TE-5, or TE-8 cells, but it is still possible that DKK1 expression is induced by Wnt signaling in S2-CP8 cells. However, in S2-CP8/ΔFOXM1 BS cells, *DKK1* mRNA was decreased while the levels of *CTNNB1* and *LEF1* mRNAs were unchanged (Fig. 8A), suggesting that DKK1 expression is differentially regulated by FOXM1 and Wnt signaling.

The upstream region (2829 bases) of the *DKK1* transcription start site was fused to the *luciferase* gene to create a full length (FL) reporter construct (Fig. 8B, left). This region contains the TCF BS, which was shown to be activated by Wnt signaling, at −96 to −71 bases upstream of the *DKK1* transcription start site [10]. Deletion of the TCF BS from the FL reporter construct (ΔTCF BS) reduced the luciferase activity induced by constitutively active β-catenin, in which the phosphorylation sites (Ser33, Ser37, Thr41, and Ser45) are mutated to alanine (β-catenin^SA^) (Fig. 8B, middle). When the FOXM1 BS (#d) was deleted from the FL reporter construct (ΔFOXM1 BS), FOXM1-dependent luciferase activity was decreased (Fig. 8B, right). Although we found a putative TCF-binding element (A/TA/TCAAAG) in #d (see Supplementary Fig. S4B), depletion of that element did not affect the β-catenin^SA^-induced luciferase activity (Fig. 8B, middle), suggesting that the possible TCF-binding element in #d is irrelevant to DKK1 expression. Moreover, FOXM1 and β-catenin^SA^ additively stimulated the luciferase activity of the FL construct (Supplementary Fig. S6D). These data support that FOXM1 binds to the upstream region of the *DKK1* gene and induces DKK1 expression independently of Wnt signaling even in cells with active Wnt signaling.

In serial sections from PDAC cases positive for both DKK1 and FOXM1, β-catenin staining was negative in 6/17 (35%) (Fig. 8C, D) and positive in 11/17 (65%) cases (Supplementary Fig. S7A). By contrast, in serial sections from ESCC cases positive for both DKK1 and FOXM1, β-catenin was negative in 23/32 (72%) (Fig. 8E, F) and positive in 9/32 (28%) cases (Supplementary Fig. S7B). The anti-β-catenin antibody was validated by compared with isotype control IgG as a primary antibody (see Supplementary Fig. S5A). A chi-square test revealed that β-catenin positivity strongly correlates with DKK1/FOXM1 positivity in PDAC compared to ESCC (Fig. 8G). These results suggest that FOXM1 promotes DKK1 expression both directly and indirectly in PDAC in cooperation with Wnt signaling and that FOXM1 primarily increases DKK1 expression independently of Wnt signaling in ESCC.

## Discussion

Based on the ability of DKK1 to inhibit the Wnt pathway, DKK1 was initially characterized as a tumor suppressor, but many studies have now linked DKK1 to cancer promotion [2, 12]. We have recently shown that DKK1 promotes cancer progression through CKAP4 (DKK1-CKAP4 signaling) [11, 21]. The present study reveals that FOXM1 acts as a transcription factor for DKK1 in cancer cells and that the DKK1-FOXM1 signaling axis creates a positive feedback loop for cancer cell proliferation (Supplementary Fig. S8). The site #d in the upstream region of the *DKK1* gene, which we identified in this study, may act as the enhancer of the *DKK1* transcription.

Both AKT and ERK are important for the expression and transcriptional activity of FOXM1 [36, 37]. Since the public database results show that the expression of DKK1 and FOXM1 are highly correlated in PDAC and ESCC, two PDAC lines (S2-CP8 and HPAF-II) and two ESCC cell lines (TE-5 and TE-8) were mainly used in this study. In these four cancer cells, FOXM1 expression is dependent upon DKK1-CKAP4 signaling, and FOXM1 transcription requires AKT and MEK activity. Since DKK1-CKAP4 signaling activates AKT, the MEK-ERK pathway would be activated by other signaling pathways in these cancer cells. CKAP4 knockdown and MEK inhibition synergistically downregulate FOXM1. Thus, inhibiting the DKK1-CKAP4 and MEK-ERK pathways simultaneously may provide a new strategy of the treatment for PDAC and ESCC expressing DKK1 and FOXM1. In addition, it has been reported that disruption of the Hippo pathway promotes FOXM1 expression in sarcoma [39]. Thus, it is intriguing to speculate that an abnormality in Hippo signaling which induces FOXM1 expression may result in an increase in DKK1 expression.

In TE-1 ESCC cells, in which CKAP4 expression is low compared to other cells, DKK1 knockdown does not affect FOXM1 expression, but FOXM1 knockdown inhibits DKK1 expression. Therefore, FOXM1 does not always require DKK1-CKAP4 signaling for its expression, but FOXM1 acts as a transcription factor for DKK1. Immunohistochemical and cell biological studies reveal that DKK1 expression correlates with FOXM1 expression but not β-catenin expression in ESCC. Our reporter assay showed that FOXM1-binding site exists ~2000 bases upstream of the DKK1 transcription start site. Taken together with the results of our immunohistochemical studies, which shows that around 70% of DKK1/FOXM1 double positive ESCC tumors are negative for β-catenin in the same tumor lesions, these results suggest that FOXM1 mainly controls DKK1 expression in ESCC.

In contrast, it has been reported that activation of Wnt signaling may initiate pancreatic cancer [40]. Indeed, S2-CP8 cells and HPAF-II cells express β-catenin in the nucleus and cytoplasm, and ~80% of PDAC cases positive for both DKK1 and FOXM1 express β-catenin in the same tumor legions. It is possible that activation of Wnt signaling, due to RNF43/ZNFR3 mutations or Wnt ligand expression [41, 42], induces DKK1 expression in PDAC. In addition, FOXM1 interacts with β-catenin and promotes β-catenin/TCF4-dependent transcription in glioma cells [38]. Indeed, β-catenin forms a complex with FOXM1 in S2-CP8 cells but not in HPAF-II, TE-5, or TE-8 cells. Taken together, these results suggest that FOXM1 expression in some PDAC cases may partly induce DKK1 expression via Wnt signaling and also support that FOXM1 is able to induce DKK1 expression directly, even in PDAC with active Wnt signaling.

In vitro and in vivo studies confirm the positive feedback loop between DKK1 and FOXM1 promotes cancer cell proliferation, and our clinical data also indicates that PDAC and ESCC cases that simultaneously expressed DKK1 and FOXM1 show poor clinical prognosis. Clinical outcomes of PDAC cases which positively expressing both proteins are not statistically significant in current study, which may be presumed due to the limited case number, but public database certainly indicates that expression of both proteins are poor prognostic factor in PDAC. Thus, the simultaneous expression of DKK1 and FOXM1 via positive feedback loop may be a remarkable hallmark of aggressive PDAC and ESCC.

It has been recently shown that CKAP4 targeting therapy via monoclonal antibody may be a novel therapeutic strategy for various cancers in which the DKK1-CKAP4 signaling axis is activated [24]. Inhibition of FOXM1 function reduces colorectal and lung tumor growth [43, 44]. Therefore, FOXM1 may be a molecular target which present a synergistic clinical effect for cancers which express it as well as DKK1 and CKAP4.

## Materials and methods

### Materials and chemicals

All cell lines, antibodies, and other chemicals used in this study are shown in Supplementary Table S5 and S6, respectively. The target sequences for the shRNA and siRNA experiments are shown in Supplementary Table S7. The primers used for quantitative PCR or Chromatin immunoprecipitation (ChIP) are shown in Supplementary Table S8 and S9, respectively.

### Patients and immunohistochemical studies of DKK1 and FOXM1

Tissue samples were obtained with informed consent from 38 newly diagnosed and previously untreated PDAC patients (median age of 70 with a range of 47–87 years) and 82 ESCC patients (median age of 71 with a range of 35–87 years) who underwent surgical resection at Osaka University hospital between October 2001 and July 2017. PDAC patients were newly diagnosed and previously untreated, and their tumors were pathologically diagnosed. Samples from 19 PDAC patients and 73 ESCC patients used in this study were also examined in our previous studies [21–23].

For immunohistochemical (IHC) study of DKK1 and FOXM1 expression, tumors in which positive staining covered 5–20% of the total area were classified as DKK1- or FOXM1- low expression, and tumors in which positive staining covered >20% of the total area were classified as DKK1- or FOXM1- high expression. Tumors in which the positively stained area covered >5% of the total area were classified as DKK1- or FOXM1- positive. Tumors in which >25% of the total area was positive for either nuclear or cytoplasmic β-catenin were classified as β-catenin positive. Serial sections of specimens were used to evaluate co-expression of DKK1 and FOXM1 (Figs. 6 and 7) and triple expression of DKK1, FOXM1, and β-catenin (Fig. 8 and Supplementary Figs. S5 and S7) in tumors. At least three investigators assessed the sections independently in a blinded fashion.

### Clinical data analyses using open sources

Data of *DKK1* and *FOXM1* mRNA expression in various organs and cancer tissues were obtained from The Genotype-Tissue Expression (GTEx) projects and The Cancer Genome Atlas (TCGA), respectively. The UCSC Xena online database (https://xenabrowser.net) was used to analyze those public data resources. The correlation between *DKK1* and *FOXM1* mRNA expression levels was analyzed by Pearson’s correlation coefficient analysis. Co-expression analysis using PDAC [33], ESCC [34], and EAC [35] datasets were obtained from ‘R2: Genomics Analysis Platform (http://r2.amc.nl)’ and visualized using Graphpad Prism. *P* values and *r* values were calculated using Prism. The correlation of overall survival rates with *FOXM1* or *DKK1* mRNA expression in the TCGA pancreatic cancer dataset was analyzed using OncoLnc (http://www.oncolnc.org). Positive expression of *FOXM1* or *DKK1* was defined based on the top 80% of the value of *FOMX1* or *DKK1* mRNA expression.

### Generation of cells lacking the FOXM1 binding site of the *DKK1* gene

The target sequence for the human *FOXM1* binding site of *DKK1*, 5ʹ- CAGATTTCCTAGTACACTGA -3ʹ and 5ʹ- CAAAAAAAATCCATTGCCTG -3ʹ, were designed with the help of CRISPR Genome Engineering Resources (https://zlab.bio/guide-design-resources) [45]. A plasmid expressing hCas9 and sgRNA sequences targeting the FOXM1 binding site of *DKK1* was prepared by ligating the oligonucleotides into the *Bbs*I site of pX330 (Addgene plasmid #48138; Addgene, Cambridge, MA), and the plasmid was transfected along with a Blasticidin S resistance plasmid into S2-CP8 cells using Viafect reagent (Promega, Madison, WI) according to the manufacturer’s instructions. The day after transfection, cells were dissociated into single cells and replated at a low density. Blasticidin S (10 µg/ml) was added one day after replating to select cells lacking the FOXM1 binding site (S2-CP8/ΔFOXM1 BS cells). The cells were allowed to grow until single cells formed colonies, which became visible after 7-14 days. Single colonies were picked, mechanically disaggregated, and replated into individual wells of 24-well plates. Colonies were amplified and analyzed by Sanger sequencing to identify mutant clones.

### Statistics

All experiments were repeated at least three times and the results are expressed as means ± S.D. Statistical analyses were performed using JMP version 11 and SAS version 9.4 (SAS Institute. Inc., Cary NC). Means and medians of continuous outcome variables were tested using the Student’s *t* test and Mann–Whitney U test, respectively. The cumulative probabilities of overall survival and relapse-free survival were computed using the Kaplan-Meier method; and the Generalized Wilcoxon test was used to assess their statistical significance. *P*-values less than 0.05 were considered statistically significant. Western blotting data are representative of at least three independent experiments.

### Study approval

The protocol for human specimens was approved by the ethical review board of the Graduate School of Medicine, Osaka University, Japan (No. 13455 and 17160), following the Declaration of Helsinki. All studies were performed in accordance with the Committee guidelines and regulations. Written informed consent was obtained from all patients. All protocols used for animal experiments in this study were approved by the Animal Research Committee of Osaka University, Japan (No. 21-048-1).

## Supplementary information

Supplementary infomation
