# Supplementary material for: The Dickkopf1 and FOXM1 positive feedback loop promotes tumor growth in pancreatic and esophageal cancers
Source: Oncogene. 2021 Jun 11;40(26):4486–502. doi: 10.1038/s41388-021-01860-z (PMC8249240; doi:10.1038/s41388-021-01860-z)
Supplement: Supplementary file 1 — Supplementary infomation [file 41388_2021_1860_MOESM1_ESM.pdf]

Supplementary Information

The Dickkopf1 and FOXM1 positive feedback loop promotes tumor growth in pancreatic and esophageal cancers.

Hirokazu Kimura[1*], Ryota Sada[1*], Naoki Takada[1*], Akikazu Harada[1], Yuichiro Doki[2], Hidetoshi Eguchi[2], Hideki Yamamoto[1], Akira Kikuchi[1#]

* These authors contributed equally to this work.

[1]Departments of Molecular Biology and Biochemistry, Osaka University, 2-2 Yamadaoka, Suita 565-0871, Japan. [2]Departments of Gastroenterological Surgery, Graduate School of Medicine, Osaka University, 2-2 Yamadaoka, Suita 565-0871, Japan.

#Correspondence author. Department of Molecular Biology and Biochemistry, Graduate School of Medicine, Osaka University

Phone: 81-6-6879-3410. Fax: 81-6-6879-3419. E-mail: akikuchi@molbiobc.med.osaka-u.ac.jp

The authors declare no competing financial interests.

Materials and chemicals

TE-5 cells were authenticated in February 2017 using short tandem repeat analysis, and authentication of the other cell lines and mycoplasma testing were not performed. Anti-CKAP4 polyclonal and monoclonal antibodies were generated as previously described [1, 2]. Generation of knockout (KO) cells using the CRISPR/Cas9 system was performed as previously described [2-4]. T7-FOXM1b was provided by Dr. Pradip Raychaudhuri (University of Illinois College of Medicine, Chicago, IL). pCW57.1-FOXM1c was purchased from Addgene (Watertown, MA). pRP-U6-*scramble* gRNA-CBh-hCas9-CMV-EGFP-Bsd was packaged by and purchased from VectorBuilder (Cyagen).

RNA sequencing

Library preparation was performed using a TruSeq stranded mRNA sample prep kit (Illumina, San Diego, CA) according to the manufacturer's instructions. The mRNA samples underwent whole transcriptome sequencing using the Illumina HiSeq 2500 platform in a 75-base single-end mode. Illumina Casava (ver.1.8.2) software was used for base calling. Sequenced reads were mapped to the human reference genome sequences (hg19) using TopHat (ver. 2.0.13) in combination with Bowtie2 (ver. 2.2.3) and SAMtools (ver. 0.1.19). The number of fragments per kilobase pairs of exon per million mapped fragments (FPKMs) was calculated using Cufflinks (ver. 2.2.1).

Chromatin immunoprecipitation (ChIP) assay

For DNA-protein cross-linking, S2-CP8 cells in culture were incubated with growth medium containing 1% formaldehyde for 20 minutes. The cells were lysed with buffer (50 mM Tris/HCl pH 8.0, 10 mM EDTA, and 0.5% sodium dodecyl sulfate (SDS)) and sonicated to shear the DNA. ChIP dilution buffer (16.7 mM Tris/HCl pH 8.0, 167 mM NaCl, 1.2 mM EDTA, and 1.1% Triton X-100) supplemented with protease inhibitors and pre-cleared with Protein A Agarose/salmon sperm DNA (Millipore, Billerica, MA, USA) was added to the sheared chromatin samples. Pre-cleared samples were then incubated with Protein A Agarose/salmon sperm DNA and 200 ng of negative control IgG, anti-FOXM1 antibody, or anti-histone H4 antibody at 4°C overnight. Precipitates were washed once with high salt buffer (20 mM Tris/HCl pH 8.1, 500 mM NaCl, 0.1% SDS, 1% TritonX-100, and 2 mM EDTA), once with LiCl buffer (10 mM Tris/HCl pH 8.1, 0.25 M LiCl, 1 mM EDTA, 1% deoxycholic acid, and 1% Nonidet P-40), and three times with TE buffer (10 mM Tris/HCl pH 8.0 and 1 mM EDTA). The eluates obtained via elution buffer (1% SDS and 100 mM $NaHCO_3$) were incubated for 4 hours at 65°C to revert DNA-protein cross-links. Next, DNA was extracted by incubation in buffer containing proteinase K (a final concentration of 0.7 g/ml) for 1 hour at 45°C. Purified DNA was used as a PCR template.

To precisely determine the putative FOXM1 binding site(s) in the *DKK1* upstream region, FOXM1-binding consensus motifs were separated into 6 clusters, #a to #f, and ChIP PCR was performed using specific couples of forward and reverse primers for each site (Supplementary Table S9). Forward and reverse primers for *AURKB* were as follows: 5′ -CCATCCTGCCTTGAAACCCA-

3′ and 5′ -AGCAAGTTACAGGCAGGAACT-3′.

Reporter gene assay

A reporter gene assay was performed using the Nano-Glo® Dual-Luciferase® Reporter Assay System (Promega) as previously described [5]. HEK293T cells (3 x 10^5 cells/well in a 6-well plate) were transfected with pGL4.27[*luc2P/minP/Hygro*] (Promega) containing genomic regions from −2885 to -56 from the transcription start site (TSS) of *DKK1* or its deletion mutants, in which the genomic region of –2050 to –1986 was deleted (FOXM1 binding site deletion: ΔFOXM1 BS) or the genomic region of –96 to –71 was deleted (TCF binding site deletion: ΔTCF BS) (1 μg), pNL1.1.PGK[*Nluc/PGK*] (Promega) (3.125 ng) and CSII-CMV-GFP-MCS-IRES2-Bsd (empty vector), CSII-CMV-FLAG-FOXM1c-IRES2-Bsd, or pCGN-HA/β-catenin^{SA}. Total amounts of transfected plasmid DNA were normalized to the same amount complemented with empty vector for each condition. 48 hours after transfection, the cells were lysed and the firefly luciferase activity was measured using ONE-Glo™ reagent (Promega). NanoDLR™ Stop & Glo® Reagent was then added to quench the firefly signal and measure NanoLuc® luciferase activity to standardize the transfection efficiency. For each sample, firefly luciferase measurements were normalized to those of NanoLuc® luciferase, and the results are expressed as fold-increase compared to the level measured in cells transfected without CSII-CMV-FLAG-FOXM1c-IRES2-Bsd or pCGN-HA/β-catenin^{SA}. Each reporter assay condition had at least three biological replicates and at least three technical replicates were performed per biological replicate.

Immunoprecipitation assay

Cells were lysed in a 60-mm diameter dish in 500 μl of Nonidet P-40 (NP-40) buffer (20 mM Tris-HCl pH 8.0, 10% glycerol, 137 mM NaCl, and 1% NP-40) with protease inhibitors (10 μg/ml leupeptin, 20 μg/ml aprotinin, and 1 mM phenylmethanesulfonyl fluoride). After centrifugation, the lysates were incubated with primary antibodies and 40 μl of a 50% slurry of protein G-Sepharose beads (GE Healthcare) for 1 hour at 4°C. After washing three times with NP-40 buffer, the precipitates were probed with the indicated antibodies.

Three-dimensional cell proliferation assay

Three-dimensional (3D) cell proliferation assays were performed as previously described [1, 4] with modification. To analyze growth of the spheres, wild-type (WT) S2-CP8 cells, S2-CP8/ΔFOXM1 BS cells, S2-CP8/ΔFOXM1 BS cells stably expressing DKK1-FLAG, S2-CP8/shDKK1 cells, and S2-CP8/shFOXM1 cells were seeded in Matrigel (BD Biosciences, San Jose, CA). Forty μl of Matrigel was mounted on a round coverslip and incubated for 30 minutes at 37 °C to solidify the gel. Cells (2×10^4) suspended in DMEM containing 1% bovine serum albumin and 2% Matrigel were added onto the solidified Matrigel and cultured for 5 days.

Xenograft tumor assay

Xenograft tumor assay was performed as previously described [1] with modification. Six-week-old male BALB/cAJcl-nu immunodeficient mice (CLEA, Tokyo, Japan) were anesthetized with a combination of medetomidine (0.3 mg/kg body weight), midazolam (4 mg/kg), and butorphanol (5.0 mg/kg). The mice then received a dorsal subcutaneous injection of S2-CP8 cells or S2-CP8/ΔFOXM1 BS cells (3×10^6 cells) suspended in 100 μl of PBS. The immunodeficient mice were then sacrificed at 21 days after transplantation. Xenograft tumors of which weight was less than 0.1 g, thereby being regarded as not engrafted, and tumors which contents were spilled due to necrosis and difficult to their weighs accurately, were excluded from analysis. The areas containing transplanted cells were measured and weighed. Tumor volumes were calculated using the following formula: (major axis) × (minor axis) × (minor axis)/2. No randomization was used to allocate mice to either cell lines. No blinding was done.

Immunocytochemistry

Cells were fixed with phosphate-buffered saline (PBS) containing 4% paraformaldehyde (PFA) and then permeabilized in PBS containing 0.2% (wt/vol) Triton X-100 and 2 mg/ml bovine serum albumin (BSA) for 10 minutes. The cells were then blocked in blocking buffer (PBS containing 2 mg/ml BSA). Next, the samples were incubated with primary antibodies diluted in PBS for 1 hour, washed three times with PBS, and then stained with secondary antibodies (conjugated to Alexa Fluor 488, 546 Invitrogen, Carlsbad, CA) diluted in PBS for 1 hour. After washing, the samples were covered with

PBS containing 50% glycerol. Samples were viewed and analyzed using an LSM880 laser scanning microscope.

Plasmid construction and lentivirus production

The lentivirus vectors expressing cDNAs were constructed by inserting the cDNAs into CSII-CMV-MCS-IRES2-Bsd provided by Dr. H. Miyoshi (RIKEN BioResource Center, Ibaraki, Japan) or pLVSIN-CMV Puro Vector (Takara Bio Inc., Shiga, Japan). To construct the lentivirus vectors harboring shRNA, an oligo DNA fragment containing the H1 promoter and the shRNA were cloned into CS-RfA-EVBsd using Gateway technology (Invitrogen), or MISSION TRC shRNAs (shDKK1-2: TRCN0000033385, shCKAP4-2: TRCN0000123295, shFOXM1-1: TRCN0000015543, shFOXM1-2: TRCN0000015544, Sigma Aldrich, St Louis, MO) were used. The lentivirus particles were generated by transfection of lentivirus vectors harboring cDNA or shRNA and the packaging vectors, pCAG-HIV-gp and pCMV-VSV-G-RSV-Rev, into X293T cells using FuGENE HD transfection reagent (Promega).

Other

The generation of S2-CP8/DKK1 KO cells and S2-CP8/CKAP4 KO cells by CRISPR/Cas9 was performed as previously described [4]. Control S2-CP8 cells in Fig. 1A and Fig. 3C were generated by transfection with the empty viral vector. To generate S2-CP8, HPAF-II, Capan-1, TE-1, TE-5, and

TE-8 cells stably expressing proteins, parental cells (5×10^4 cells/well in a 12-well plate) were transduced with the constructed lentivirus and selected using Blasticidin S or puromycin. Knockdown of protein expression by siRNA and quantitative PCR were performed as previously described [1, 6-8]. For Western blotting, clathrin and HSP90 were used as loading controls. Sample sizes were chosen empirically based on optimizing the maximum number of samples per independent experiment while considering the 3Rs (reduction/refinement/replacement) of animal research.

References

1 Kimura H, Fumoto K, Shojima K, Nojima S, Osugi Y, Tomihara H *et al*. CKAP4 is a Dickkopf1 receptor and is involved in tumor progression. J Clin Invest 2016; 126: 2689-2705.

2 Kimura H, Yamamoto H, Harada T, Fumoto K, Osugi Y, Sada R *et al*. CKAP4, a DKK1 receptor, is a biomarker in exosomes derived from pancreatic cancer and a molecular target for therapy. Clin Cancer Res 2019; 25: 1936-1947.

3 Fujii S, Shinjo K, Matsumoto S, Harada T, Nojima S, Sato S *et al*. Epigenetic upregulation of ARL4C, due to DNA hypomethylation in the 3'-untranslated region, promotes tumorigenesis of lung squamous cell carcinoma. Oncotarget 2016; 7: 81571-81587.

4 Sada R, Kimura H, Fukata Y, Fukata M, Yamamoto H, Kikuchi A. Dynamic palmitoylation controls the microdomain localization of the DKK1 receptors CKAP4 and LRP6. Sci Signal 2019; 12: eaat9519.

5 Andrilenas KK, Ramlall V, Kurland J, Leung B, Harbaugh AG, Siggers T. DNA-binding landscape of IRF3, IRF5 and IRF7 dimers: implications for dimer-specific gene regulation. Nucleic Acids Res 2018; 46: 2509-2520.

6 Yamamoto H, Komekado H, Kikuchi A. Caveolin is necessary for Wnt-3a-dependent internalization of LRP6 and accumulation of β-catenin. Dev Cell 2006; 11: 213-223.

7 Matsumoto S, Fujii S, Sato A, Ibuka S, Kagawa Y, Ishii M *et al*. A combination of Wnt and growth factor signaling induces Arl4c expression to form epithelial tubular structures. EMBO J 2014; 33: 702-718.

8 Fujii S, Matsumoto S, Nojima S, Morii E, Kikuchi A. Arl4c expression in colorectal and lung cancers promotes tumorigenesis and may represent a novel therapeutic target. Oncogene 2015; 34: 4834-4844.

Supplementary Table 1. Relationship between DKK1 and FOXM1 expression and clinicopathological characteristics of pancreatic ductal adenocarcinoma cases (n = 38)

Parameters	DKK1 positive (n = 29)	DKK1 negative (n = 9)	*P* value
Age (year)	71 (47-86)	66 (51-87)	0.47
Sex (male/female)	17/12	5/4	1.00
pT (1/2/3)	2/1/26	2/1/6	0.26
pN (0/1)	9/20	5/4	0.25
ly (0/1/2)	10/12/7	4/4/1	0.69
v (0/1/2/3)	15/8/5/1	7/0/2/0	0.30
ne (0/1/2/3)	2/9/15/3	4/4/1/0	0.018

Parameters	FOXM1 positive (n = 33)	FOXM1 negative (n = 5)	*P* value
Age (year)	69 (47-86)	80 (59-87)	0.19
Sex (male/female)	18/15	4/1	0.37
pT (1/2/3)	3/2/28	1/0/4	0.67
pN (0/1)	11/22	3/2	0.34
ly (0/1/2)	11/16/6	3/0/2	0.12
v (0/1/2/3)	19/7/6/1	3/1/1/0	0.98
ne (0/1/2/3)	4/11/15/3	2/2/1/0	0.35

Parameters	DKK1 and FOXM1 positive (n = 27)	Others (n = 11)	*P* value
Age (year)	71 (47-86)	66 (51-87)	0.50
Sex (male/female)	15/12	7/4	0.65
pT (1/2/3)	2/1/24	2/1/8	0.46
pN (0/1)	8/19	6/5	0.27
ly (0/1/2)	10/12/5	4/4/3	0.82
v (0/1/2/3)	15/7/4/1	7/1/3/0	0.53
ne (0/1/2/3)	2/8/14/3	4/5/2/0	0.045

T1, Tumor limited to the pancreas, 2 cm or less in greatest dimension. T2, Tumor limited to the pancreas, more than 2 cm in greatest dimension. T3, Tumor extends beyond the pancreas but without involvement of the celiac axis or the superior mesenteric artery. N0, No regional lymph node metastasis. N1, Regional lymph node metastasis. ly0, No lymphatic vessel invasion. ly1, Mild lymphatic vessel invasion. Ly2, Moderate lymphatic vessel invasion. v0, No venous invasion. v1, Mild venous invasion. v2, Moderate venous invasion. v3, High venous invasion. ne0, No perineural invasion. ne1, Mild perineural invasion. ne2, Moderate perineural invasion. ne3, High perineural invasion.

Supplementary Table 2. Relationship between DKK1 and FOXM1 expression and clinicopathological characteristics of esophageal squamous cell carcinoma cases (n = 82)

Parameters	DKK1 positive (n = 46)	DKK1 negative (n = 36)	*P* value
Age (year)	67 (35-83)	68 (50-79)	0.56
Sex (male/female)	41/5	31/5	0.68
Location (Ce/Ut/Mt/Lt/Ae)	1/5/25/13/2	1/8/16/9/2	0.72
pT (Tis/1/2/3)	0/4/13/29	2/7/9/18	0.17
pN (0/1/2/3)	9/16/14/7	16/13/5/2	0.04
Parameters	FOXM1 positive (n = 71)	FOXM1 negative (n = 11)	*P* value
Age (year)	67 (35-83)	68 (58-82)	0.43
Sex (male/female)	63/8	9/2	0.51
Location (Ce/Ut/Mt/Lt/Ae)	2/12/33/20/4	0/0/1/8/2/0	0.65
pT (Tis/1/2/3)	1/9/20/41	1/2/2/6	0.41
pN (0/1/2/3)	22/25/17/7	3/4/2/2	0.85
Parameters	DKK1 and FOXM1 positive (n = 40)	Others (n = 42)	*P* value
Age (year)	67 (35-83)	68 (50-82)	0.45
Sex (male/female)	36/4	36/6	0.55
Location (Ce/Ut/Mt/Lt/Ae)	1/4/21/12/2	1/9/20/10/2	0.69
pT (Tis/1/2/3)	0/3/12/25	2/8/10/22	0.20
pN (0/1/2/3)	9/14/12/5	16/15/7/4	0.33

Ce, Cervical esophagus, Ut, Upper thoracic esophagus, Mt, Middle thoracic esophagus, Lt, lower thoracic esophagus, Ae, Abdominal esophagus, Tis, Tumor in situ (non-invasive), T1, Tumor invades to lamina propria, muscularis mucosae, or submucosa. T2, Tumor invades muscularis propria. T3, Tumor invades adventitia. T4, Tumor invades adjacent structures.N0, No regional lymph node metastasis. N1, Metastasis in 1-2 regional lymph nodes. N2, Metastasis in 3-6 regional lymph nodes. N3, Metastasis in 7 or more regional lymph nodes.

Supplementary Table 3. Univariate analysis of relapse free survival of ESCC cases by Cox's proportional hazard model

Parameters	Number	Hazard ratio	95% CI	*P* value
Sex (male/female)	72/10	0.819	0.370-2.170	0.66
Age (≥65years/<65years)	52/30	1.021	0.544-1.983	0.95
pT (2-3/Tis-2)	69/13	1.491	0.639-4.351	0.38
pN (1-3/0)	57/25	2.491	1.163-6.170	0.017
DKK1 expression (positive/negative)	46/36	2.256	1.171-4.619	0.014
FOXM1 expression (positive/negative)	71/11	1.801	0.723-6.046	0.22
DKK1/FOXM1 expression (both positive/others)	40/42	2.249	1.195-4.380	0.011

Supplementary Table 4. Multivariate analysis of overall survival of ESCC by Cox's proportional hazard model

Parameters	Number	Hazard ratio	95% CI	*P* value
pN (1-3/0)	57/25	2.499	1.165-6.193	0.017
DKK1/FOXM1 expression (both positive/others)	25/57	1.962	1.029-3.668	0.041

Supplementary Table 5. List of cell lines used in this study

Cell line	Species	Source	Time	Culture medium
S2-CP8	Human PDAC	Cell Resource Center for Biomedical Research, Institute of Development, Aging and Cancer Tohoku University	April 2014	DMEM with 10% FBS
HPAF-II	Human PDAC	American Type Culture Collection	July 2017	DMEM with 10% FBSwith 10% FBS
Panc02.13	Human PDAC	American Type Culture Collection	February 2019	RPMI-1640 with 10 Units/ml human recombinant insulin and 15% FBS
Capan-1	Human PDAC	Provided by Dr. Y. Kanai (Osaka University, Suita, Japan)	September 2018	DMEM with 10% FBS
TE-1	Human ESCC	Riken Bioresource Center Cell Bank (Tsukuba, Japan)	January 2015	RPMI-1640 with 10% FBS
TE-5	Human ESCC	Riken Bioresource Center Cell Bank (Tsukuba, Japan)	November 2008	RPMI-1640 with 10% FBS
TE-8	Human ESCC	Riken Bioresource Center Cell Bank (Tsukuba, Japan)	January 2015	RPMI-1640 with 10% FBS
TE-10	Human ESCC	Riken Bioresource Center Cell Bank (Tsukuba, Japan)	January 2015	RPMI-1640 with 10% FBS
Lenti-X™ 293T	Human embryonic kidney	Takara Bio Inc.	October 2011	DMEM with 10% FBS
HEK293T	Human embryonic kidney	American Type Culture Collection	April 2002	DMEM with 10% FBS

PDAC, pancreatic ductal adenocarcinoma; DMEM, Dulbecco's modified Eagle's medium; FBS, fetal bovine serum; ESCC, esophageal squamous cell carcinoma.

Supplementary Table 6. List of antibodies and other chemicals used in this study

Antigen	Company	Catalog #	Purpose	Dilution ratio
Clathrin	BD Biosciences	610500	WB	1:1000
HSP90	BD Biosciences	610418	WB	1:1000
DKK1	Cell Signaling Technology	48367S	WB	1:200
DKK1	R&D Systems	MAB10962	IHC	1:200
AKT	Cell Signaling Technology	9272	WB	1:1000
pAKT (S473)	Cell Signaling Technology	9271	WB	1:1000
FOXM1	Cell Signaling Technology	20459S	WB, IP, and ChIP	1:500 (WB, IP), 0.2 μg/ml (ChIP)
FOXM1	Santa Cruz Biotechnology	Sc-376471	IHC	1:100
Erk	Cell Signaling Technology	4695S	WB	1:1000
pErk	Cell Signaling Technology	4370S	WB	1:1000
β-catenin	BD Biosciences	610154	WB, IP, IF, and IHC	1:1000 (WB, IP) 1:500 (IF) 1:500 (IHC)
Ki-67	Cell Signaling Technology	9449S	IF	1:800
IgG	Cell Signaling Technology	2729S	ChIP, IHC	0.2 μg/ml (ChIP) 1:100 (IHC)

Product	Company	Catalog #
AKT inhibitor VIII	Merck Millipore	124018
PD0325901	FUJIFILM Wako Pure Chemical Corporation	162-25291
Nano-Glo® Dual-Luciferase® Reporter Assay System	Promega	N1610

WB, western blotting; ChIP, Chromatin immunoprecipitation; IHC, immunohistochemistry.

Supplementary Table 7. Target sequences for shRNA and siRNA used in this study

Gene	Sequence
Human *CKAP4* #1 (shRNA)	GCAGATTAACCTCAGAAAT
Human *CKAP4* #2 (shRNA)	GCAGGATTTGAAAGCCTTAAA
Human *CKAP4* #1 (siRNA)	GCTGCAGAAACTCCAGAAT
Human *CKAP4* #2 (siRNA)	GCAGATTAACCTCAGAAAT
Human *CTNNB1* #1 (siRNA)	CCCACTAATGTCCAGCGTT
Human *DKK1* #1 (shRNA)	GGATCTCTTGGAATGACAA
Human *DKK1* #2 (shRNA)	CGGTTCTCAATTCCAACGCTA
Human *DKK1* #1 (siRNA)	GCTCTCATGGACTAGAAAT
Human *DKK1* #2 (siRNA)	CCTGGAGTGTAAGAGCTTT
Human *FOXM*1 #1 (shRNA)	GCAAGAAGAAATCCTGGTTAA
Human *FOXM1* #2 (shRNA)	GCCCAACAGGAGTCTAATCAA
Control (shRNA)	GTGCGTTGCTAGTACCAAC
Control (siRNA)	CAGTCGCGTTTGCGACTGG

Supplementary Table 8. Primer sequences for quantitative PCR used in this study

Gene	Forward	Reverse
Human *AURKB*	GGGAGAGCTGAAGATTGCTG	GGCGATAGGTCTCGTTGTGT
Human *BIRC5*	GGACCACCGCATCTCTACAT	TCTCCGCAGTTTCCTCAAAT
Human *DKK1*	AAGCGCCGAAAACGCTGCAT	GATCTTGGACCAGAAGTGTC
Human *FOXM1*	CTGTTCAAAATGCCCCAAGT	TGCTGTGATGATGCTGTGAA
Human *FOXM1a*	TGGGGAACAGGTGGTGTTTGG	CCTCCTCAGCTAGCAGCACT
Human *FOXM1b*	CCAGGTGTTTAAGCAGCAGA	TCCTCAGCTAGCAGCACCTTG
Human *FOXM1c*	CAATTGCCCGAGCACTTGGAATCA	TCCTCAGCTAGCAGCACCTTG
Human *CTNNB1*	TAGAAACAGCTCGTTGTACCGCTGGGACCT	GCACTGCCATTTTAGCTCCTTCTTGATGTAAT
Human *AXIN2*	CTGGCTCCAGAAGATCACAAAG	CATCCTCCCAGATCTCCTCAAA
Human *LEF1*	GGGACCCTCCTACTCGAGTT	ATGTGTGACGGGTGTGATCC
Human *UBC*	CCTGGTGCTCCGTCTTAGAG	TTCCCAGCAAAGATCAACC
Human *GAPDH*	TCCTGCACCACCAACTGCTT	TGGCAGTGATGGCATGGAC

Supplementary Table 9. Primer sequences for ChIP PCR used in this study

Amplified cluster	Forward	Reverse
#a	CTCCACCGCCCAAATAAAT	TCTCATTGACTTTTAAAACAGTTTGG
#b	GGCTGCAGTTAACCACGATT	TTGTCTGTCTCCCTCATGGA
#c	CATGTGTTAGCATGCTGTTCC	TAGGGAACCCTGCCTCCTAT
#d	GCCCCAGGCAATGGATTTTT	TGCGTTAAGCACCTTCAGTG
#e	TCTCCCTTTTCCAAACTCACA	GAGGGGCACAGTGAATACAAAT
#f	CCCCCTTCATGTACACAAACA	ACCGCGGCTGCCTTTATAC

Kimura et al., Supplementary Figure S1

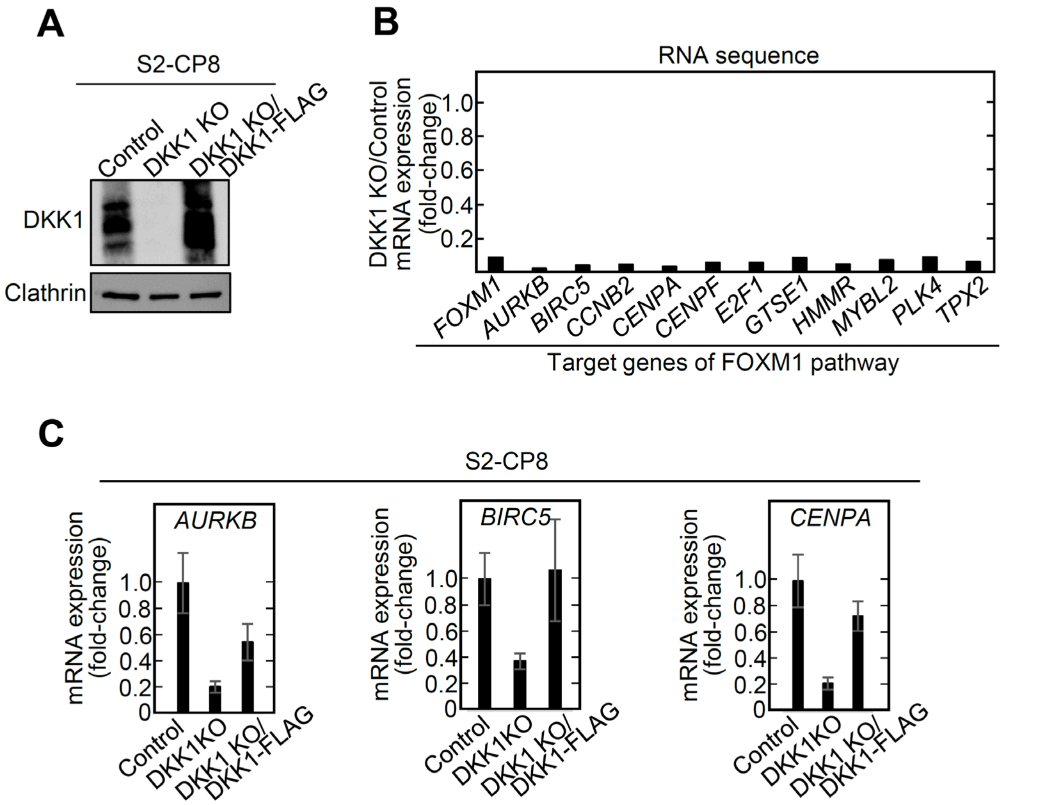

Supplementary Fig. S1. DKK1 KO decreases FOXM1-dependent gene expression.

(A) Lysates from control S2-CP8 cells, S2-CP8/DKK1 KO cells, and S2-CP8/DKK1 KO/DKK1-FLAG cells were probed with the indicated antibodies. Clathrin was used as a loading control.

(B) Relative mRNA levels of the FOXM1 pathway genes in S2-CP8/DKK1 KO cells are shown as fold-changes compared to control S2-CP8 cells.

(C) The mRNA levels of *AURKB*, *BIRC5,* and *CENPA* in the S2-CP8 cells used in Supplemental Fig. S1A were measured by quantitative RT-PCR, and the relative mRNA levels were normalized to *GAPDH*. The results are shown as fold-changes compared to control cells and are expressed as means ± S.D. from three independent experiments.

Kimura et al., Supplementary Figure S2

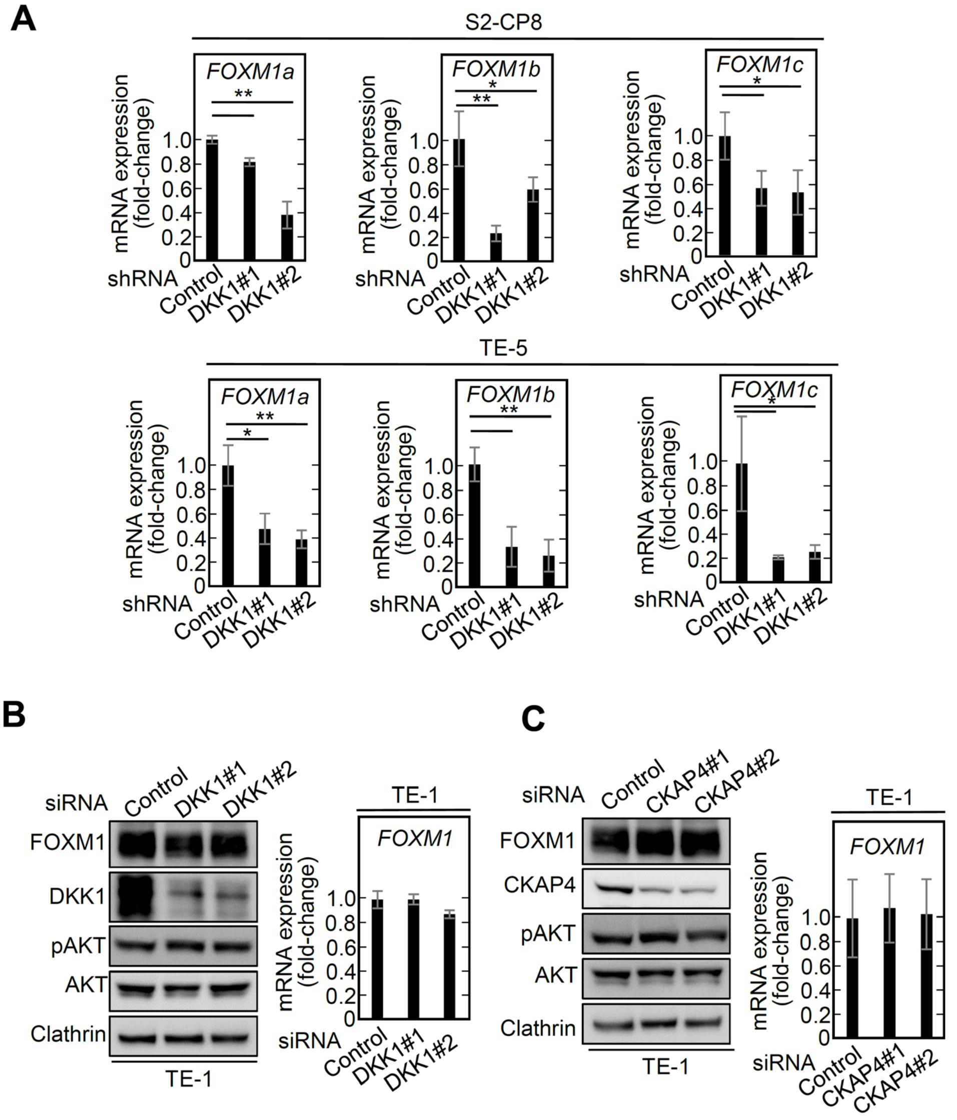

Supplementary Fig. S2. DKK1 KD decreases FOXM1 expression.

(A) The mRNA levels of *FOXM1a*, *FOXM1b*, and *FOXM1c* in S2-CP8 cells (top panel) and TE-5 cells (bottom panel) were measured by quantitative RT-PCR. Relative mRNA levels were normalized to *GAPDH* and are shown as fold-changes compared to control shRNA-expressing cells. Results are shown as means ± S.D. from three independent experiments.

(B and C) Left panels: lysates from TE-1 cells transfected with control (scramble) siRNA or DKK1 siRNAs (B) and control (scramble) siRNA or CKAP4 siRNAs (C) were probed with the indicated antibodies. Clathrin was used as a loading control. Right panels: the mRNA levels of *FOXM1* in TE-1 cells transfected with control (scramble) siRNA or DKK1 siRNAs (B) and control (scramble) siRNA or CKAP4 siRNAs (C) were measured by quantitative RT-PCR and normalized to *GAPDH.* The results are shown as fold-changes compared to control siRNA transfected cells and are expressed as means ± S.D. from three independent experiments. *, $P < 0.05$; **, $P < 0.01$ (Student's t test).

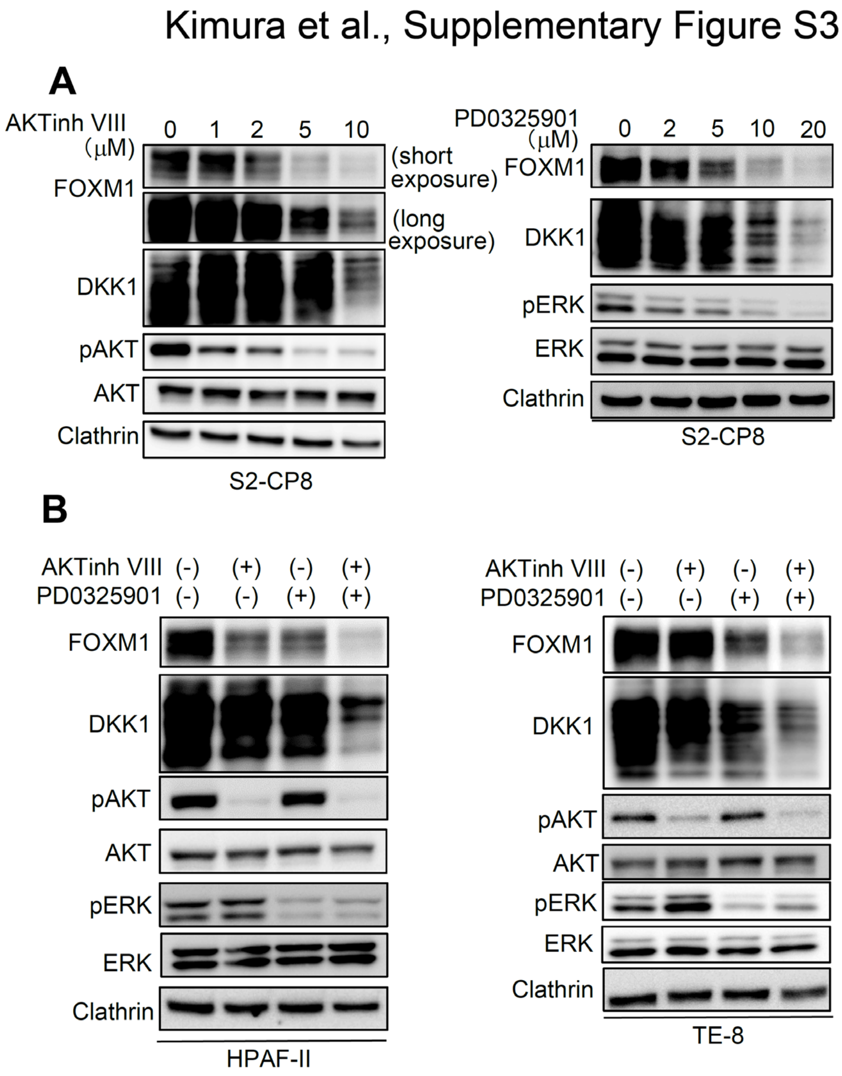

Supplementary Fig. S3. Inhibition of AKT and MEK suppresses the expression of FOXM1 and DKK1.

(A) S2-CP8 cells were treated with the indicated concentration of AKT inhibitor VIII (left panel) or PD0325901 (right panel) for 48 hours, and the lysates were probed with the indicated antibodies. Clathrin was used as a loading control.

(B) HPAF-II cells (left panel) and TE-8 cells (right panel) were treated with AKT inhibitor VIII (5 μM), PD0325901 (5 μM), or both inhibitors for 48 hours, and the lysates were probed with the indicated antibodies. Clathrin was used as a loading control.

Kimura et al., Supplementary Figure S4

A

sgRNA	Guide sequence (5’→3’)
FOXM1 binding site deletion 5’ targeting	CAGATTTCCTAGTACACTGA
FOXM1 binding site deletion 3’ targeting	CAAAAAAAATCCATTGCCTG

B

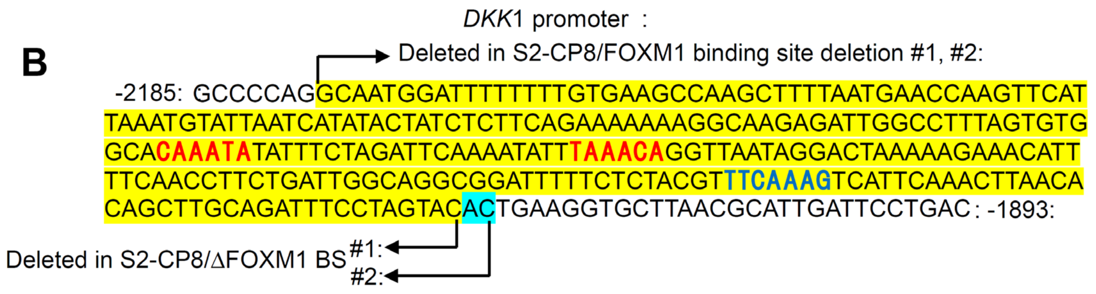

Supplementary Fig. S4. Knockout of the FOXM1 biding site of the *DKK1* gene.

(A) The sgRNA designed for the 5’- and 3’- target sequences of the FOXM1 binding site of the *DKK1* gene.

(B) The genomic sequences of the upstream promoter region of the *DKK1* gene. The region which was depleted in the S2-CP8/ΔFOXM1 BS cells #1 and #2 was confirmed via sequence analysis, and the depleted regions are high-lightened in yellow and light blue, respectively. The putative FOXM1 binding site and the putative TCF-binding element are shown in red characters and blue characters, respectively.

Kimura et al., Supplementary Figure S5

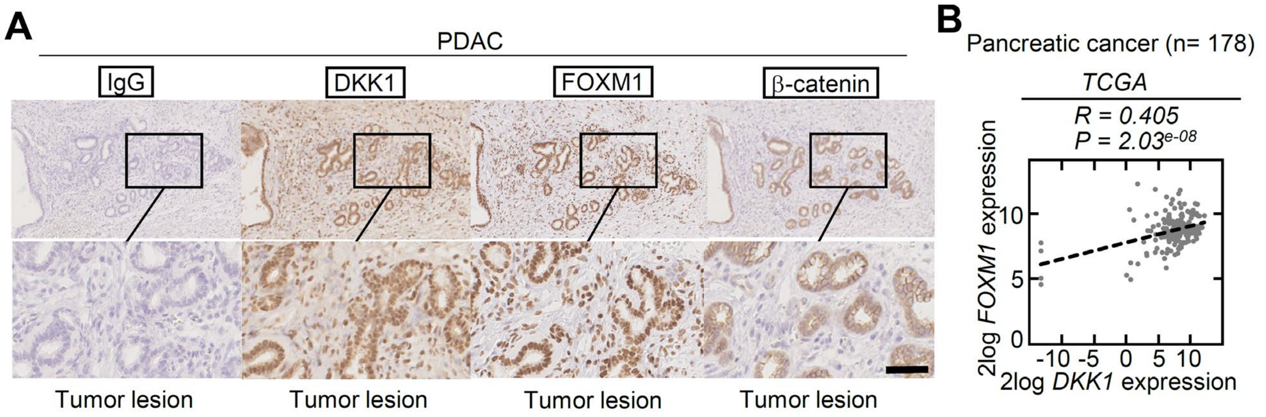

Supplementary Fig. S5. Specificity of the antibodies used in immunohistochemical study.

(A) Serial sections of PDAC tissues were stained with isotype control IgG or antibodies for DKK1, FOXM1, or β-catenin used in Figs. 6, 7, and 8 and Supplementary Fig. S7 and hematoxylin. Black boxes show enlarged images. Scale bar, 50 µm.

(B) Scatter plot showing the correlation between *DKK1* (X-axis) and *FOXM1* (Y-axis) mRNA expression in pancreatic cancers. The data was obtained from R2: Genomics Analysis and Visualization Platform. The dotted black line indicates linear fit. *R* indicates the Pearson's correlation coefficient.

Kimura et al., Supplementary Figure S6

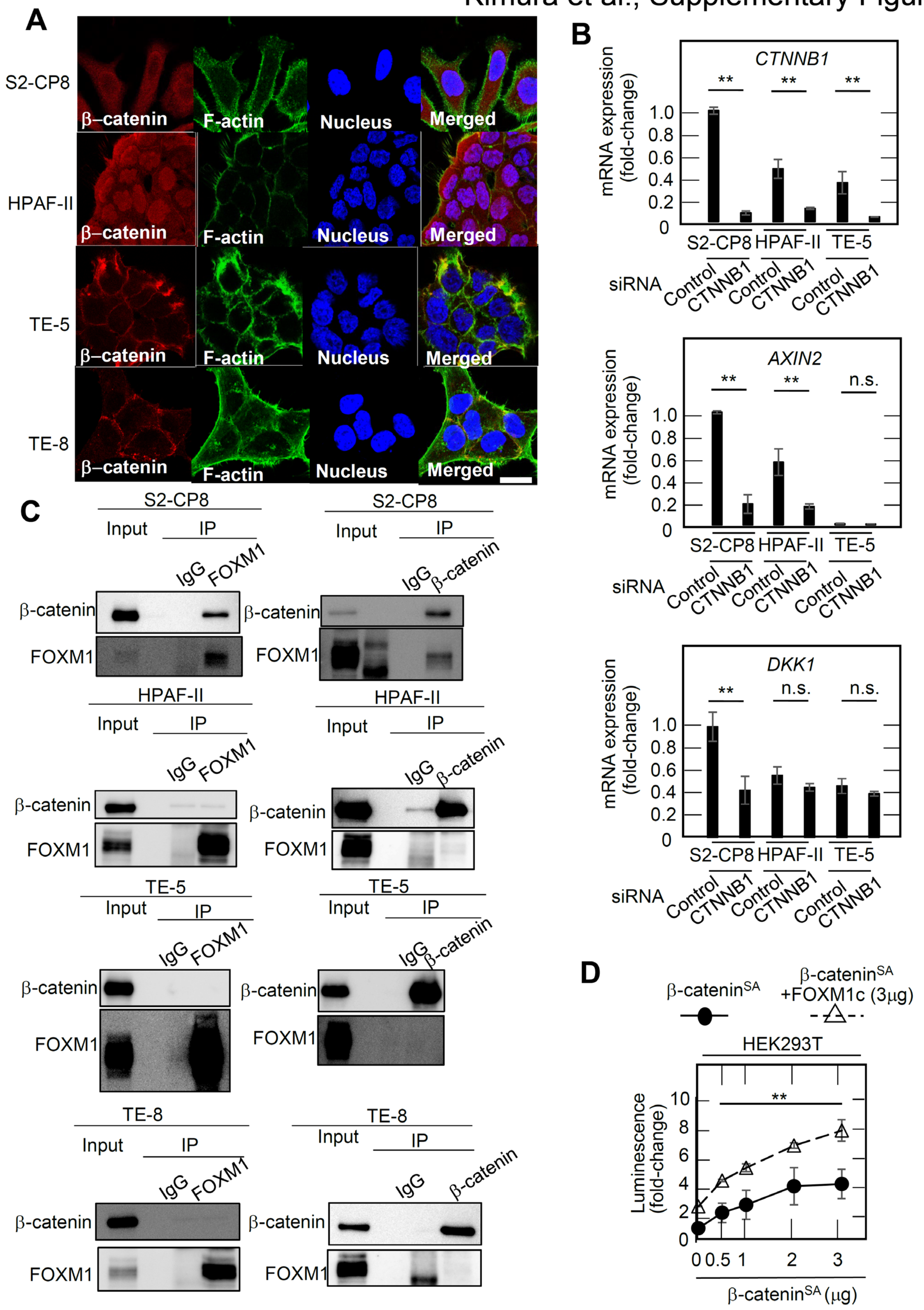

279

280

Supplementary Fig. S6. FOXM1 induces DKK1 expression dependent upon Wnt/β-catenin signaling in pancreatic cancer cells.

(A) S2-CP8, HPAF-II, TE-5, and TE-8 cells were stained with anti-β-catenin (red) and Alexa488-conjugated phalloidin to visualize F-actin (green), and DRAQ5 was used to visualize the nucleus (blue). Scale bar, 20 μm.

(B) The mRNA levels of *CTNNB1*, *AXIN2*, and *DKK1* in S2-CP8, HPAF-II, and TE-5 cells transfected with control or CTNNB1 siRNAs were measured by quantitative RT-PCR and normalized to *GAPDH.* The results are shown as fold-changes compared to the control siRNA-transfected S2-CP8 cells and are expressed as means ± S.D. from three independent experiments.

(C) Lysates (input) of S2-CP8, HPAF-II, TE-5, and TE-8 cells were immunoprecipitated with anti-FOXM1, anti-β-catenin, or control IgG antibodies. The immunoprecipitates (IP) were probed with anti-FOXM1 and anti-β-catenin antibodies. The second lane from the left of the right panel using S2-CP8 cells is a blank lane, but non-specific bands are stained.

(D) HEK293T cells were transfected with the FL reporter construct and the indicated amounts of HA-β-catenin^{SA} expression vector with 3 μg of FLAG-FOXM1c expression vector (the dotted line and open triangle) or control vector (the solid line and closed circles), and luciferase activities were measured. The results are shown as fold-changes compared to cells without FOXM1c and β-catenin^{SA} expression. The results are expressed as means ± S.D. from three independent experiments. **, $P < 0.01$ (Student's t test).

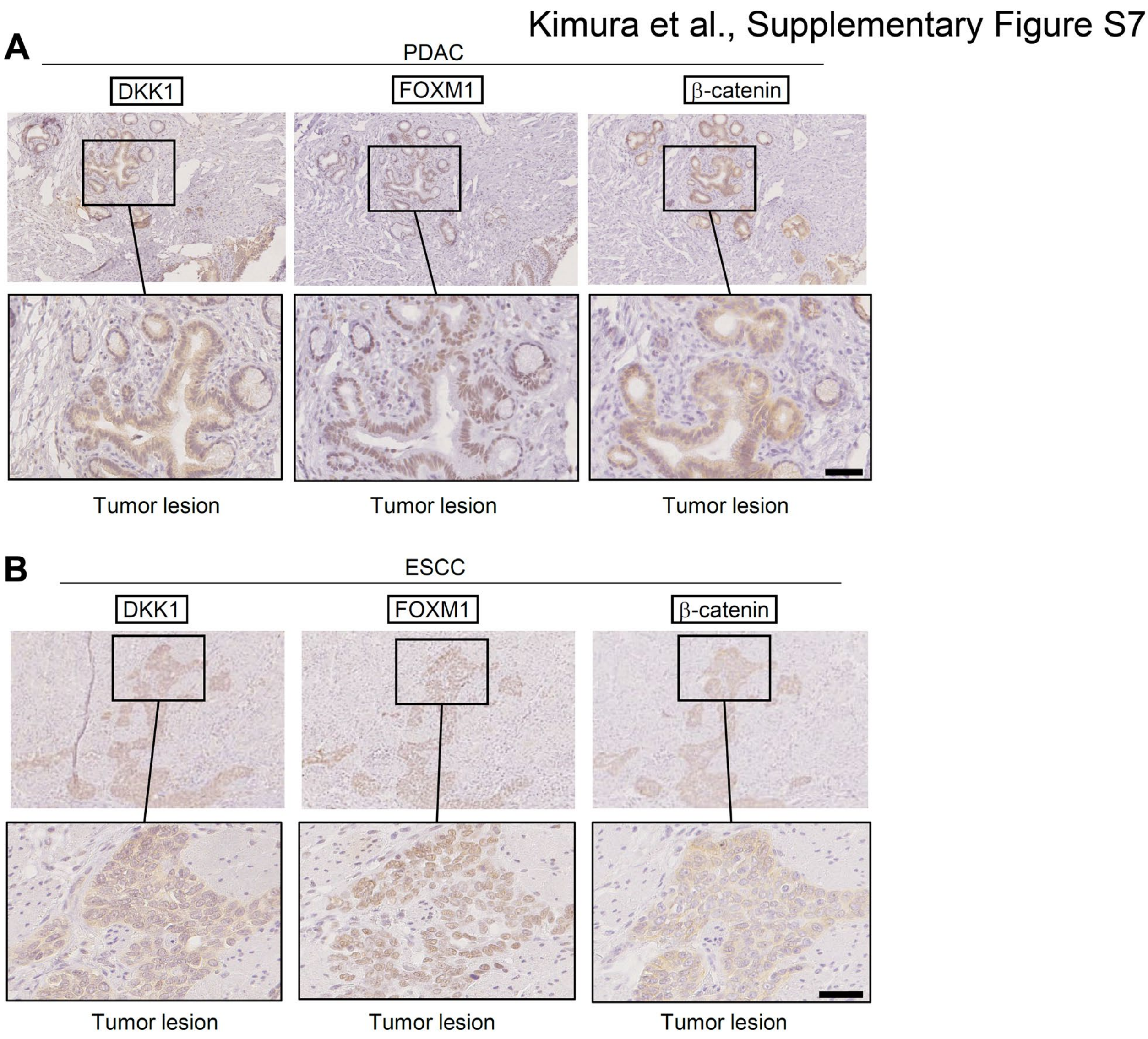

Supplementary Fig. S7. β-catenin is co-expressed with FOXM1 and DKK1 in PDAC and ESCC.

Representative images of serial sections of DKK1, FOXM1, and β-catenin-triple positive cases from PDAC tissues (n = 11) (A) and ESCC tissues (n = 23) (B) used in Fig. 8 are shown. Black boxes show enlarged images. Scale bars, 50 μm.

Kimura et al., Supplementary Figure S8

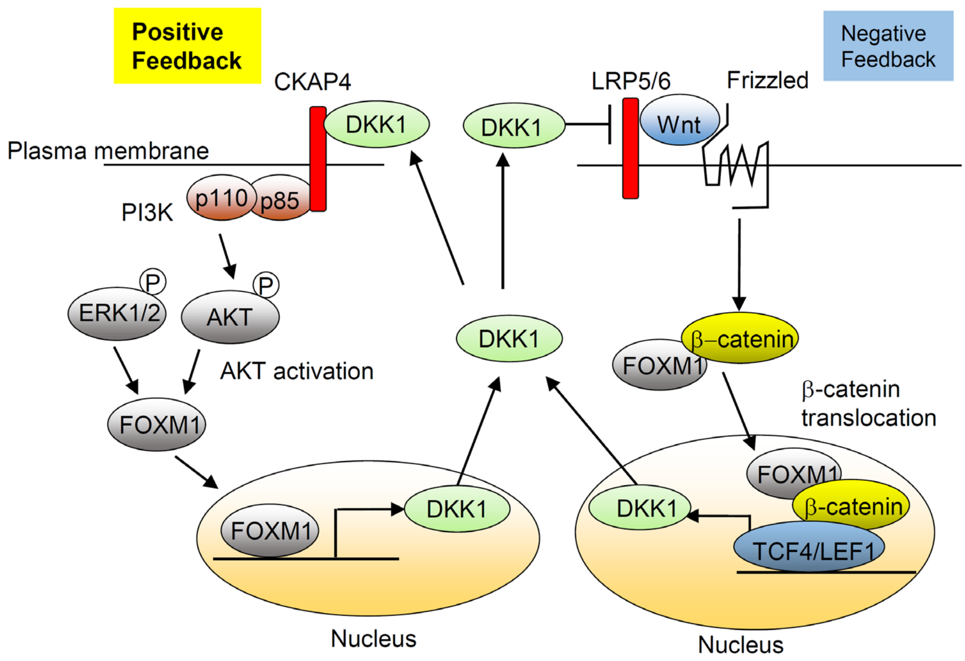

Supplementary Fig. S8. A schematic model of the positive feedback loop of DKK1-FOXM1 signaling.

The activation of ERK and AKT pathways in cancer cells induces FOXM1 expression. FOXM1 directly binds to the enhancer region of the *DKK1* gene and stimulates DKK1 expression. Then, secreted DKK1 binds to CKAP4 in the cell surface membrane, which activates PI3K-AKT pathway to further increase DKK1 expression. Thus, DKK1 and FOXM1 enhance their expression mutually in a positive feedback loop and promote tumor cell proliferation. FOXM1 also binds to β-catenin, and the complex enhances TCF4-dependent transcription, resulting in DKK1 production. Thus, aberrant activation of ERK, AKT, and Wnt pathways may lead to tumorigenesis through the expression of FOXM1 and DKK1.

DKK1 was originally identified as a negative regulator of Wnt signaling by binding to the Wnt co-receptor LRP5/6. However, when the Wnt signaling is activated downstream of Wnt receptors (Frizzled and LRP5/6), for instance due to the β-catenin mutation, the negative regulation by DKK1 may not work. In this case, DKK1 produced by the activation of Wnt signaling could lead to cancer cell proliferation through CKAP4.
